# rAAV-mediated multi-miRNA delivery targeting MAPK pathway suppresses laser-induced choroidal neovascularization in mice

**DOI:** 10.1016/j.omtn.2026.103101

**Published:** 2026-09-16

**Authors:** Jung Woo Han, Jin Young Yang, Yeji Kim, Sumin An, Eunjae Yoo, In Hwan Park, Sang-Won Lee, Hyun-Ji Lee, Tae Geol Lee, Wook Hyun Jung, Hyo Song Park, Jin Ha Kim, Jun Sub Choi, Hun Soo Chang, Tae Kwann Park

**Affiliations:** 1 Department of Ophthalmology, Soonchunhyang University Bucheon Hospital, Bucheon, Republic of Korea; 2 Department of Ophthalmology, Soonchunhyang University College of Medicine, Cheonan 31151, Republic of Korea; 3 Laboratory of Molecular Therapy for Retinal Degeneration, Soonchunhyang University Bucheon Hospital, Bucheon, Republic of Korea; 4 Department of Interdisciplinary Program in Biomedical Science, Graduate School, Soonchunhyang University, Asan, Republic of Korea; 5 Nanobio Measurement Group, Korea Research Institute of Standards and Science, Daejeon 34113, Republic of Korea; 6 Department of Advanced Bioconvergence, University of Science and Technology, Daejeon 34113, Republic of Korea; 7 Department of Microbiology, College of Medicine, Soonchunhyang University, Cheonan, Republic of Korea

**Keywords:** MT: delivery strategies, age-related macular degeneration, choroidal neovascularization, MAPK signaling, miRNA therapy, rAAV, gene therapy, angiogenesis, fibrosis, EMT, oxidative stress

## Abstract

Age-related macular degeneration (AMD) is a leading cause of irreversible vision loss, with neovascular AMD (nAMD) characterized by choroidal neovascularization (CNV) and subretinal fibrosis. Although anti-VEGF therapy is the current standard, frequent injections and limited efficacy necessitate alternative treatments. The mitogen-activated protein kinase (MAPK) pathway contributes to AMD by promoting oxidative stress-induced apoptosis, inflammation, and angiogenesis. We evaluated the therapeutic potential of MAPK-targeting microRNAs (miRNAs) delivered via recombinant adeno-associated virus (rAAV). Two rAAV2.7m8-based strategies were developed: a MAPK-miR mixture (three individually packaged miRNAs: hsa-miR-122-5p, let-7a-5p, and miR-26a-5p) and an MAPK-miR tandem (all three miRNAs in one vector under separate U6 promoters). Both formulations effectively inhibited MAPK activation in oxidative stress-induced RPE cells and laser-induced CNV mouse models. Treatment reduced CNV leakage and angiogenesis, as confirmed by fluorescein angiography and histology. The MAPK-miR mixture showed enhanced efficacy in suppressing epithelial-mesenchymal transition (EMT) and subretinal fibrosis. This study demonstrates the therapeutic benefit of rAAV-mediated multi-miRNA delivery targeting MAPK signaling in AMD. Sustained expression from rAAV vectors may overcome the limitations of anti-VEGF therapy, offering a novel gene therapy approach for nAMD. Further optimization and safety evaluation are warranted for clinical translation.

## Graphical abstract

**Figure:**
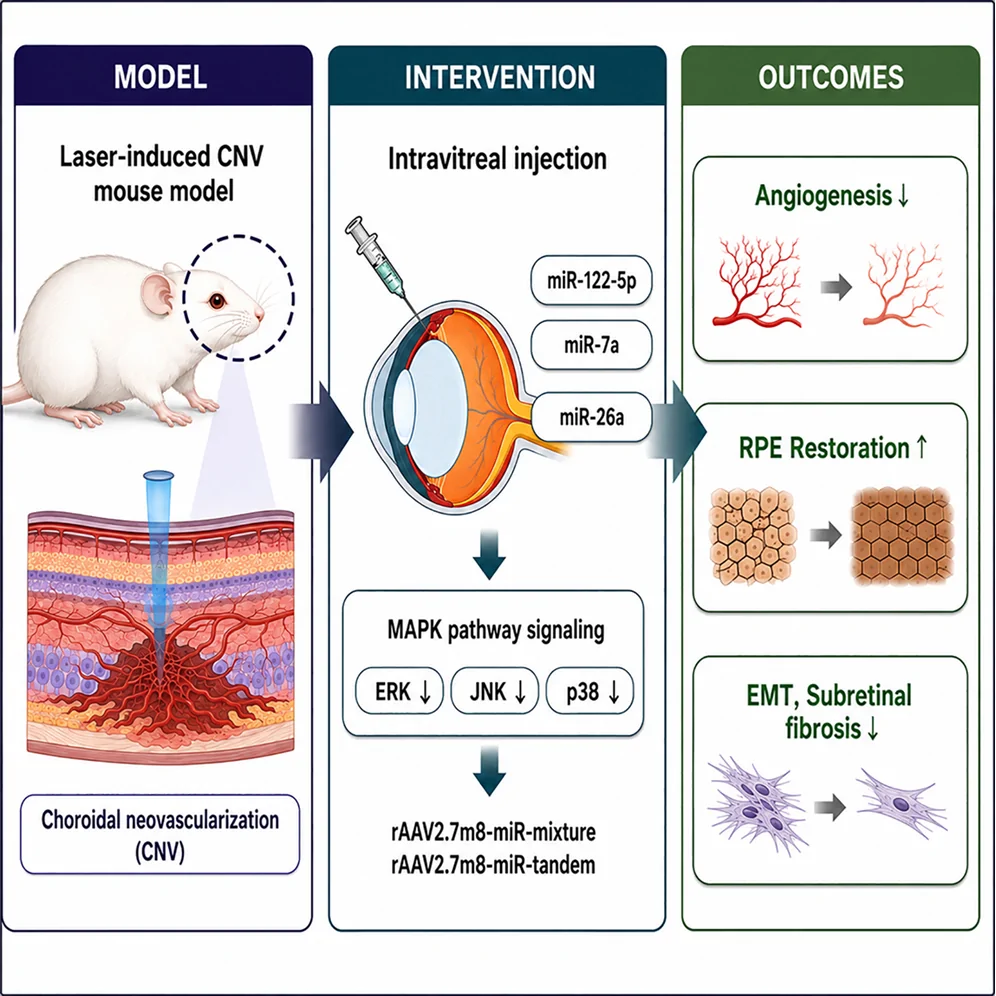

## Teaser

AAV2.7m8 delivery of three MAPK-targeting miRNAs suppresses MAPK signaling, reduces oxidative stress-induced RPE apoptosis, and attenuates CNV in a laser-induced AMD model, with the miRNA mixture showing superior efficacy. This multi-miRNA strategy represents a promising alternative or adjunct to anti-VEGF therapy.

## Introduction

Age-related macular degeneration (AMD) is a leading cause of irreversible central vision loss in the elderly.^1^ AMD is typically classified into two main forms: geographic atrophy (aAMD), commonly referred to as “dry” AMD, and neovascular AMD (nAMD), or “wet” AMD.^2^^,^^3^ Although nAMD accounts for only 10%–15% of all AMD cases, it is responsible for the majority of severe vision loss due to choroidal neovascularization (CNV), which can result in secondary subretinal hemorrhage, photoreceptor cell death, and ultimately, permanent subretinal fibrovascular scarring.^4^^,^^5^^,^^6^ Current treatments primarily involve repeated intravitreal injections of anti-vascular endothelial growth factor (VEGF) antibodies every 4–12 weeks.^7^^,^^8^ However, approximately 10%–20% of nAMD patients exhibit poor or no response to anti-VEGF therapy, highlighting the urgent need for novel therapeutic approaches.^9^^,^^10^^,^^11^ Additionally, frequent intravitreal injections impose significant economic and procedural burdens, often leading to suboptimal patient compliance. Moreover, sustained VEGF inhibition has been associated with adverse effects, including increased risk of ocular complications such as endophthalmitis, intraocular inflammation, and atrophic changes,^12^ further prompting the exploration of alternative therapeutic strategies.

The etiology of AMD is multifactorial, with both genetic and environmental factors playing a role. A growing body of evidence implicates oxidative stress in the pathogenesis and progression of AMD.^13^ Reactive oxygen species (ROS) can activate several signaling pathways, including the mitogen-activated protein kinase (MAPK) cascade, which regulates key cellular processes such as proliferation, differentiation, and apoptosis.^14^ Numerous studies have shown that oxidative stress-induced degeneration of retinal pigment epithelium (RPE) cells is mediated, at least in part, by MAPK signaling.^15^^,^^16^^,^^17^ Notably, activation of MAPK components—such as extracellular signal-regulated kinase (ERK), c-Jun N-terminal kinase (JNK), and p38 MAPK—has been observed in human RPE cultures and animal models of AMD,^18^^,^^19^ underscoring the therapeutic potential of MAPK inhibition.

MicroRNAs (miRNAs) are short, noncoding RNAs approximately 22 nucleotides in length that modulate gene expression post-transcriptionally by binding to the 3′ untranslated regions (3′-UTRs) of target mRNAs, resulting in mRNA degradation or translational repression.^20^^,^^21^^,^^22^ Dysregulation of miRNA expression has been implicated in a variety of human diseases, making them attractive candidates for mutation-independent therapeutic strategies.^23^

Recent studies in animal models and humans have highlighted the significant roles of miRNAs in the development and progression of CNV.^24^ In our previous study, we demonstrated that MAPK signaling is involved in the progression of CNV.^25^ Furthermore, we showed that exosomal miRNAs derived from retinal organoids can target the MAPK signaling pathway and alleviate photoreceptor cell death in RCS rats.^26^ Based on these findings, we hypothesized that miRNAs modulating MAPK signaling could serve as potential therapeutic agents for AMD. To investigate this possibility, we performed an analysis to identify specific exosomal miRNAs associated with the MAPK pathway. From this analysis, we selected the three abundant miRNAs—hsa-miR-122-5p, hsa-let-7a-5p, and hsa-miR-26a-5p—based on their complementary and comprehensive coverage of almost all the critical genes associated with the MAPK signaling pathway^27^^,^^28^^,^^29^^,^^30^^,^^31^^,^^32^^,^^33^ as analyzed using miRTarBase version 8.0 and TarBase v8.0 integrated within miRNet (Figure S1). These miRNAs are also known to regulate multiple target mRNAs across diverse signaling pathways; however, in this study, we focused on the MAPK signaling pathway due to its established role in CNV and fibrosis. Specifically, these three miRNAs collectively target key components involved in all three major branches of MAPK signaling, including ERK, JNK, and p38 MAPK cascades. By combining these three miRNAs, we aimed to achieve simultaneous and comprehensive suppression across all branches of MAPK signaling, thereby maximizing therapeutic efficacy in the treatment of CNV in the laser-induced CNV mouse model.

We employed an adeno-associated virus serotype 2.7m8 (AAV2.7m8) vector-based approach to deliver these MAPK-related miRNAs (rAAV2.7m8-MAPK-miR) by constructing two distinct AAV formulations: one containing a mixture of individually packaged miRNAs (rAAV2.7m8-MAPK-miR mixtures) and another carrying all three miRNAs within a single vector (rAAV2.7m8-MAPK-miR tandem). These vectors were administered via intravitreal injection into the CNV mouse model to assess their ability to suppress CNV. This study demonstrates that rAAV2.7m8-mediated delivery of miRNA mixtures or tandems can effectively inhibit MAPK signaling and subsequently suppress CNV, highlighting the therapeutic potential of multi-miRNA-based gene therapy for AMD.

## Results

### Design and validation of rAAV2.7m8-MAPK-miR constructs

To evaluate the therapeutic potential of MAPK-targeting miRNAs, we designed two distinct recombinant adeno-associated virus (rAAV) vectors:rAAV2.7m8-MAPK-miR mixtures and rAAV2.7m8-miR tandem. The rAAV2.7m8-MAPK-miR mixture was generated by individually packaging hsa-miR-122-5p, hsa-let-7a-5p, and hsa-miR-26a-5p into separate AAV vectors, each driven by a U6 promoter for efficient transcription. Each miRNA was cloned in a conventional shRNA configuration designed to generate a stem-loop RNA transcript that can be processed by the endogenous RNAi machinery. The exact RNA sequences and predicted shRNA secondary structures of the expressed hairpin transcripts, including the scramble control, are provided in Figure S2. To facilitate the tracking of each miRNA, we incorporated distinct fluorescent reporter genes: mCherry for hsa-miR-122-5p, TagBFP2 for hsa-let-7a-5p, and EGFP for hsa-miR-26a-5p. These individually packaged rAAVs were then mixed in equal proportions to create the rAAV2.7m8-MAPK-miR mixture (Figure 1A).

**Figure 1:**
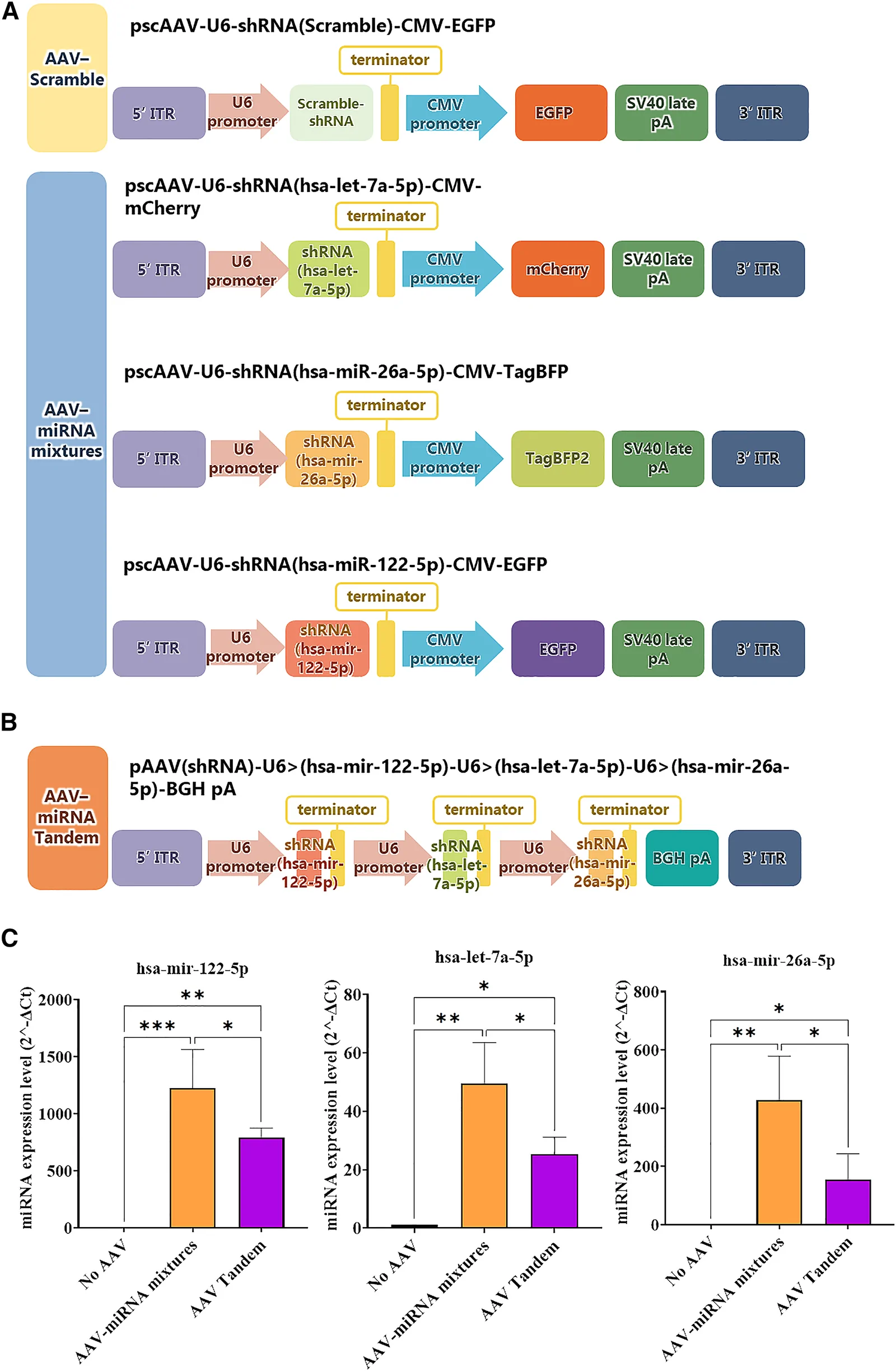
Design and validation of rAAV constructs for MAPK-targeting miRNA delivery (A) Schematic diagrams of the rAAV2.7m8 constructs. A scramble control vector (AAV-scramble) expressing a non-targeting shRNA under the U6 promoter was included as a negative control. For the miRNA mixture, three individual AAV vectors were generated, each encoding a single miRNA (hsa-let-7a-5p, hsa-miR-26a-5p, and hsa-miR-122-5p) in a short hairpin RNA (shRNA)-like structure driven by the U6 promoter. Each construct also contained a distinct fluorescent reporter gene (mCherry, TagBFP2, or EGFP) under the CMV promoter to enable visualization of transduction efficiency. These vectors were mixed in equal proportions to generate the rAAV2.7m8-MAPK-miR mixture. (B) Schematic representation of the rAAV2.7m8-MAPK-miR tandem construct. All three miRNAs were incorporated into a single vector as shRNA-like hairpin structures, each driven by an independent U6 promoter to enable separate transcription and reduce potential transcriptional interference. A BGH polyadenylation signal was included downstream. (C) Quantitative analysis using TaqMan probe-based PCR confirms significantly elevated expression levels of hsa-miR-122-5p, hsa-let-7a-5p, and hsa-miR-26a -5p in the culture media of ARPE-19 cells treated with rAAV2.7m8-MAPK-miR mixtures and rAAV2.7m8-MAPK-miR tandem compared with untreated control cells (No AAV). Statistical significance was analyzed using one-way ANOVA with post hoc comparisons. Data are represented as mean ± SEM (∗*p* < 0.05, ∗∗*p* < 0.01, ∗∗∗*p* < 0.001).

When applied to ARPE-19 cells, fluorescence imaging confirmed that mCherry, TagBFP2, and EGFP were distinctly and efficiently expressed, indicating successful transduction and expression of each miRNA construct (Figure S3A). To further validate *in vivo* transduction efficiency, retinal tissues were examined following intravitreal injection of the rAAV2.7m8-MAPK-miR mixture. In retinal cross-sections, the expression of TagBFP2, EGFP, and mCherry became detectable as early as 3 days post-injection and was maintained at 7 days, indicating successful and sustained transduction across retinal layers (Figure S3B). Consistent with these findings, RPE flat-mount analysis also demonstrated clear expression of all three fluorescent reporters at both 3 and 7 days post-injection. The merged images confirmed co-localization of the three signals within the transduced regions, further supporting successful delivery and expression of each miRNA construct *in vivo* (Figure S3C). Together, these results demonstrate efficient and stable transduction of the rAAV2.7m8-MAPK-miR mixture both *in vitro* and *in vivo*.

In contrast, the rAAV2.7m8-MAPK-miR tandem was designed to express all three miRNAs from a single vector (Figure 1B). To mitigate potential transcriptional competition and ensure independent miRNA expression, we inserted separate U6 promoters upstream of each miRNA within the same construct. The miRNAs were arranged sequentially as hsa-miR-122-5p, hsa-let-7a-5p, and hsa-miR-26a-5p, based on normalized expression levels obtained from miRNA profiling of retinal organoid secretion,^26^ reflecting a physiologically relevant expression pattern in the retina. A bovine growth hormone polyadenylation (BGHpA) signal, commonly used as a transcription termination and polyadenylation element in expression vectors, was included in the construct. This tandem-based design was intended to enable the co-expression of multiple miRNAs from a single vector. However, we did not systematically compare different vector configurations or promoter arrangements, and therefore cannot conclude whether this design provides optimized or balanced expression.

To compare the expression efficiency of the two formulations, quantitative analysis was performed using TaqMan probe-based PCR to measure the levels of the three miRNAs in the culture medium. The results demonstrated that all three miRNAs were significantly increased in both the rAAV2.7m8-miRNA mixture and the tandem-treated groups compared with control, confirming successful expression and secretion. Notably, the expression levels of all three miRNAs were significantly higher in the rAAV2.7m8-miRNA mixture-treated group compared with the rAAV2.7m8-MAPK-miR tandem-treated group (Figure 1C). The raw Ct values for all experimental groups are provided in Table S1.

### rAAV2.7m8-MAPK-miR protects ARPE-19 cells from oxidative stress-induced apoptosis via MAPK signaling inhibition

To assess the efficacy of rAAV2.7m8-MAPK-miR mixture and rAAV2.7m8-MAPK-miR tandem in an *in vitro* model, ARPE19 cells were exposed to a specific concentration of H_2_O_2_ to create a model of oxidative stress-induced damage in RPE cells. Treatment with rAAV2.7m8-MAPK-miR mixtures and rAAV2.7m8-MAPK-miR tandem significantly reduced the levels of phosphorylated MAPK pathway proteins, including *p*-JNK, p-p38, and *p*-ERK, compared with the H_2_O_2_-treated group. In contrast, the total protein levels of JNK, p38, and ERK did not show significant differences among the experimental groups (Figure S4). Accordingly, the ratios of p-JNK/JNK, p-p38/p38, and p-ERK/ERK were also significantly decreased in the rAAV2.7m8-MAPK-miR mixture- and tandem-treated groups (Figures 2A and 2B).

**Figure 2:**
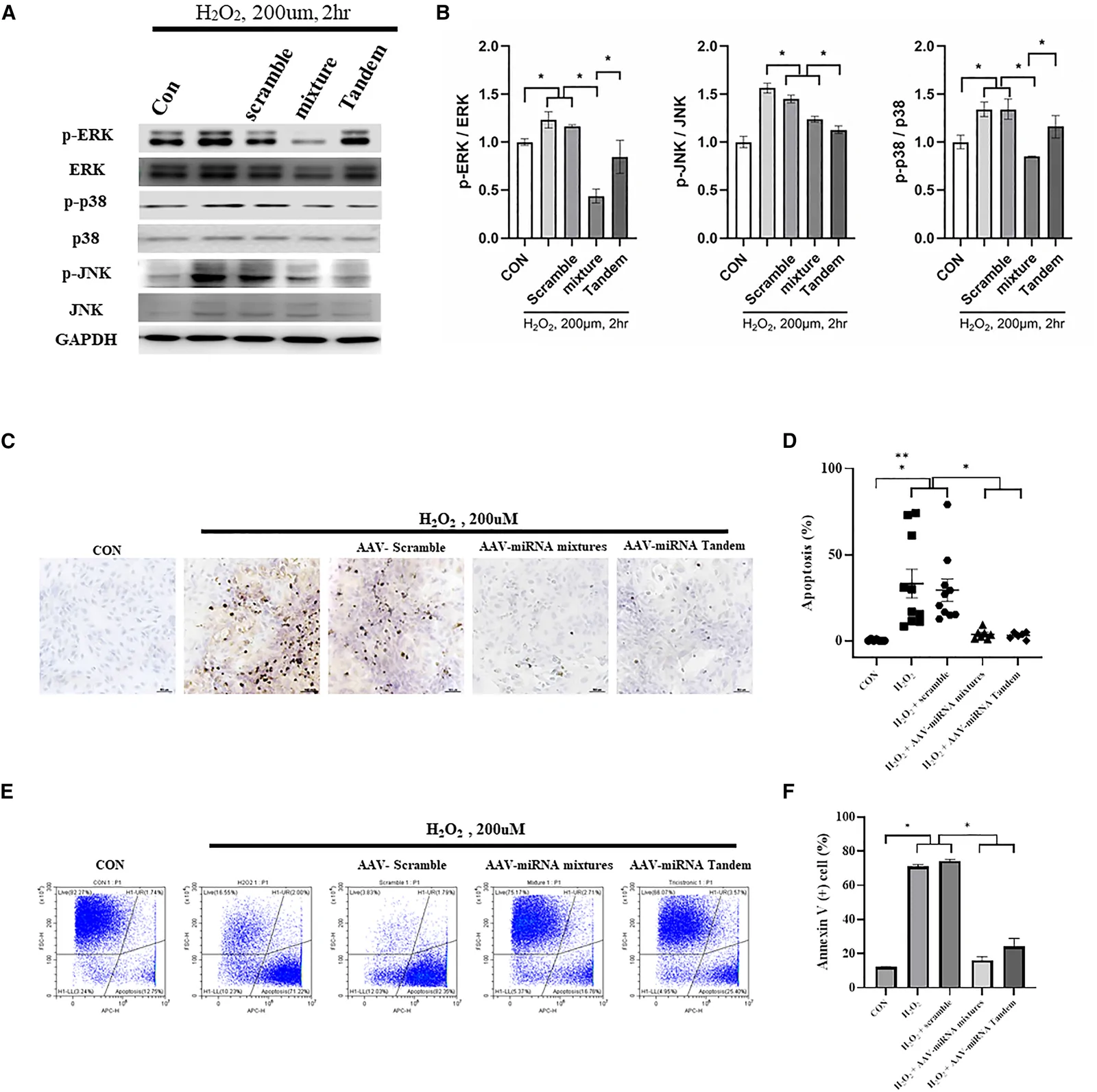
rAAV2.7m8-MAPK-miR protects ARPE-19 cells from oxidative stress-induced apoptosis through inhibition of MAPK signaling (A) Western blot analysis showing the expression levels of phosphorylated ERK (p-p44/42), JNK (*p*-JNK), and p38 MAPK (p-p38) relative to their total protein levels (p44/42, JNK, and p38) in ARPE-19 cells treated with 200 μM H_2_O_2_ for 2 h in the presence or absence of rAAV2.7m8-MAPK-miR mixtures, rAAV2.7m8-MAPK-miR tandem, or AAV-scramble. GAPDH served as a loading control. (B) Quantitative densitometric analysis of western blot data, demonstrating significant reduction of p-ERK, p-JNK, and p-p38 levels following treatment with rAAV-MAPK-miR mixtures and tandem compared with H_2_O_2_-treated cells (∗*p* < 0.05). (C) Representative images of TUNEL staining illustrating apoptotic cells (dark nuclei) after exposure to 200 μM H_2_O_2_. Cells treated with rAAV2.7m8-MAPK-miR mixtures and tandem exhibit markedly fewer apoptotic nuclei compared with the AAV-scramble group. Scale bars, 50 μm. (D) Quantification of TUNEL-positive apoptotic cells (%), confirming significant apoptosis reduction by rAAV2.7m8-MAPK-miR mixtures and tandem treatments (∗*p* < 0.05, ∗∗∗*p* < 0.001). (E) Representative flow cytometric plots of Annexin V-APC staining showing apoptotic cell populations in control, H₂O₂-treated, AAV-scramble, rAAV2.7m8-MAPK-miR mixture, and rAAV2.7m8-MAPK-miR tandem groups. (F) Quantification of Annexin V-positive cells (%), demonstrating significantly reduced apoptosis in the rAAV2.7m8-MAPK-miR mixture and tandem groups compared with the H₂O₂-treated and AAV-scramble groups (∗*p* < 0.05). Data are presented as mean ± SEM.

Subsequently, to investigate whether the cytoprotective effect of rAAV2.7m8-MAPK-miR mixtures and rAAV2.7m8-MAPK-miR tandem was involved in the regulation of apoptosis, cell apoptosis was determined with TUNEL nuclei staining and Annexin V-APC flow cytometry. The percentage of apoptosis of TUNEL-positive cells due to H_2_O_2_ treatment was significantly increased. In contrast, rAAV2.7m8-MAPK-miR mixtures and rAAV2.7m8-MAPK-miR tandem treatment significantly inhibited H_2_O_2_-induced apoptosis (Figures 2C and 2D). The anti-apoptotic effect of rAAV2.7m8-MAPK-miR mixtures and rAAV2.7m8-MAPK-miR tandem in ARPE-19 cells was further verified using Annexin V-APC flow cytometry. Compared with the control group, the percentage of apoptotic cells in the H_2_O_2_-treated group significantly increased, while both rAAV2.7m8-MAPK-miR mixtures and rAAV2.7m8-MAPK-miR tandem reduced the degree of apoptosis of ARPE-19 cells treated with H_2_O_2_ (Figures 2E and 2F). Notably, both the mixture and tandem treatments demonstrated a greater reduction in apoptosis compared with treatment with individual miRNAs—hsa-miR-122-5p, hsa-let-7a-5p, or hsa-miR-26a-5p—indicating that a combinatorial miRNA approach may offer additive protective effect against oxidative damage (Figure S5). Overall, these results suggest that both rAAV2.7m8-MAPK-miR mixtures and rAAV2.7m8-MAPK-miR tandem reduced apoptosis via MAPK signaling inhibition in an oxidative stress model of RPE Cells.

### Inhibition of MAPK pathway activation by rAAV2.7m8-MAPK-miR in laser-induced CNV

Laser-induced CNV was established in mice, and intravitreal injections were administered 5 days after photocoagulation. One week after injection, therapeutic effects were evaluated using fundus fluorescein angiography (FFA), optical coherence tomography (OCT), and immunofluorescence (IF) (Figure 3A). Immediately after laser photocoagulation, infrared (IR) imaging was used to confirm the successful induction of CNV lesions, as indicated by the presence of a characteristic bubble formation reflecting Bruch’s membrane rupture. Laser spots without bubble formation were excluded from further analysis (Figure S6).

**Figure 3:**
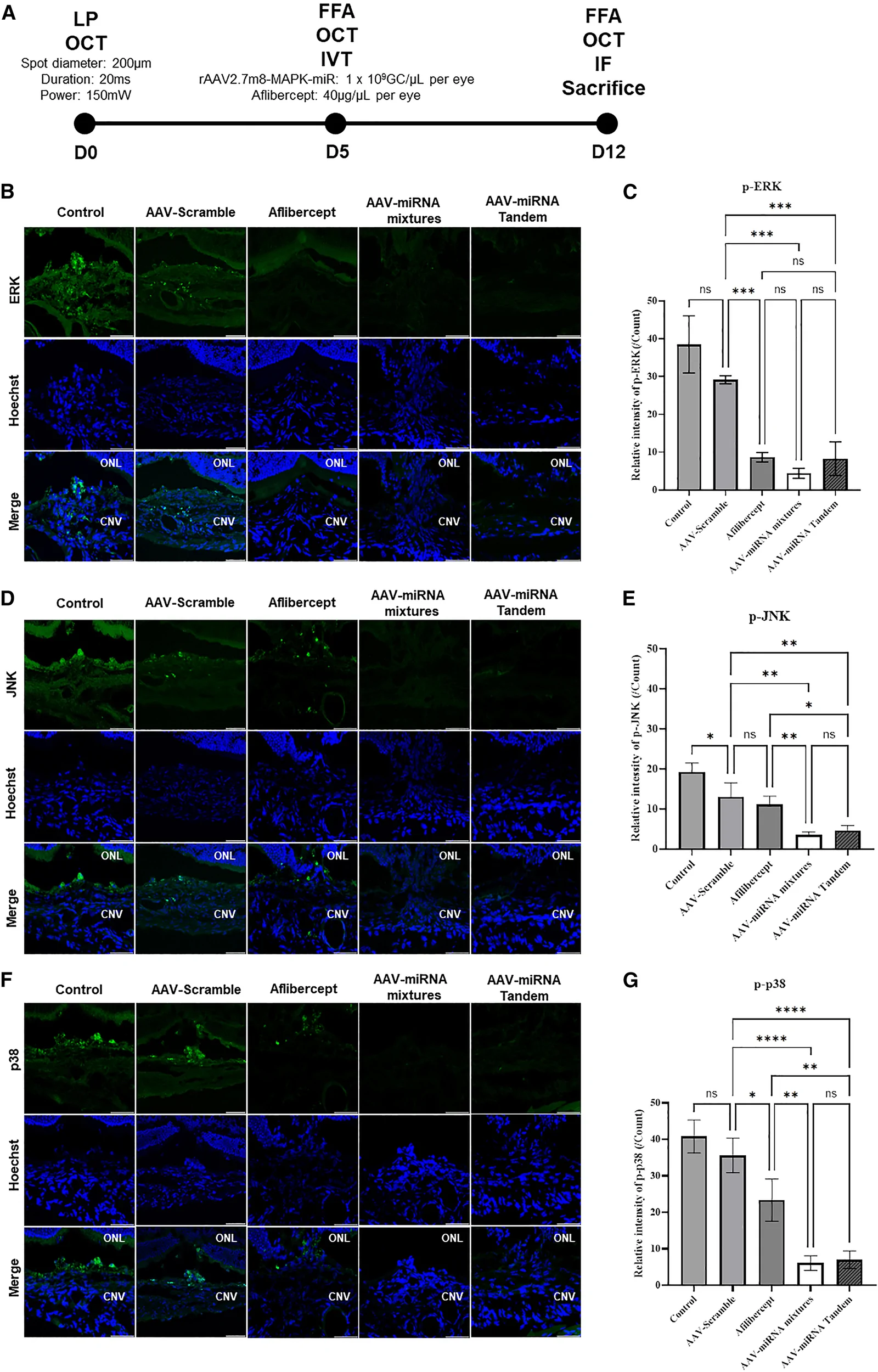
Suppression of MAPK pathway activation by rAAV2.7m8-MAPK-miR in laser-induced CNV lesions (A) Schematic timeline of the experimental design. Laser photocoagulation was performed to induce CNV, followed by intravitreal injection of AAV vectors 5 days post-laser. Retinal tissues were collected for analysis 7 days after injection. (B, D, F) Representative immunofluorescence images of phosphorylated MAPK proteins—p-ERK (B), p-JNK (D), and p-p38 (F) (green)—in retinal cross-sections from each treatment group. Nuclei were counterstained with Hoechst (blue). CNV lesions and the outer nuclear layer (ONL) are indicated. In the control (untreated) and AAV-scramble groups, strong upregulation of p-ERK, *p*-JNK, and p-p38 signals was observed within CNV regions. In contrast, treatment with rAAV2.7m8-MAPK-miR mixture and tandem markedly reduced the expression of these phosphorylated MAPK proteins. Aflibercept reduced *p*-ERK expression but showed limited effects on p-JNK and p-p38. (C, E, G) Quantitative analysis of fluorescence intensity for *p*-ERK (C), *p*-JNK (E), and p-p38 (G) within CNV regions. Statistical significance was analyzed using one-way ANOVA with post hoc comparisons (∗*p* < 0.05, ∗∗*p* < 0.01, ∗∗∗*p* < 0.001, ∗∗∗∗*p* < 0.0001; ns, not significant). Data are presented as mean ± SEM (*n* = 3–5 per group). Scale bars, 50 μm.

First, we investigate whether rAAV2.7m8-MAPK-miR could suppress activated MAPK signaling in laser-induced CNV in mice. In normal retina without CNV, phosphorylated MAPK signals, including p-ERK, p-JNK, and p-p38, were minimally detected, indicating low basal activation of the MAPK pathway (Figure S7). In contrast, the expression of *p*-ERK expression was significantly upregulated in CNV lesions. Treatment with rAAV2.7m8-MAPK-miR mixture, rAAV2.7m8-MAPK-miR tandem, and aflibercept resulted in a significant reduction of *p*-ERK expression, whereas AAV-scramble showed no such effect (Figures 3B and 3C). Additionally, the expression levels of *p*-JNK and p-p38 were also upregulated in CNV lesions. Notably, rAAV2.7m8-MAPK-miR mixture and rAAV2.7m8-MAPK-miR tandem effectively suppressed *p*-JNK and p-p38 expression, while aflibercept treatment did not, showing a pattern similar to rAAV-scramble (Figures 3D–3G).

To further validate these findings, western blot analysis was performed. Consistent with the immunostaining results, the phosphorylation levels of MAPK proteins, expressed as the ratios of phosphorylated to total protein (p-ERK/ERK, p-JNK/JNK, and p-p38/p38), were significantly increased in CNV lesions and were reduced following treatment with rAAV2.7m8-MAPK-miR mixture and tandem. In contrast, aflibercept did not show a comparable reduction in *p*-JNK/JNK ratios (Figure S8).

Taken together, these results indicate that rAAV2.7m8-MAPK-miR mixture and tandem effectively suppress MAPK pathway activation at both the tissue and protein levels, supporting their potential as therapeutic agents targeting multiple MAPK signaling pathways in CNV.

### rAAV2.7m8-MAPK-miR therapy attenuates CNV leakage and reduces structural lesions in OCT

On day 5, distinct and well-defined hyperfluorescent spots were observed within all laser photocoagulation zones, indicating the presence of CNV lesions. These hyperfluorescent spots persisted in mice injected with the control and rAAV-scramble-treated groups. In contrast, FFA images from the aflibercept-, rAAV2.7m8-MAPK-miR mixtures-, and rAAV2.7m8-MAPK-miR tandem-treated groups exhibited significant reductions in CNV leakage (Figure 4A). Quantitative analysis of FFA images revealed a reduction in the hyperfluorescent area in the aflibercept-, rAAV2.7m8-MAPK-miR mixture-, and rAAV2.7m8-MAPK-miR tandem-treated groups on day 12 compared with day 5 (*p* < 0.01, *p* < 0.0001, and *p* < 0.05, respectively) (Figure 4B). However, a statistically significant reduction in hyperfluorescent intensity was observed only in the rAAV2.7m8-MAPK-miR mixture-treated group (*p* < 0.01) (Figure 4C).

**Figure 4:**
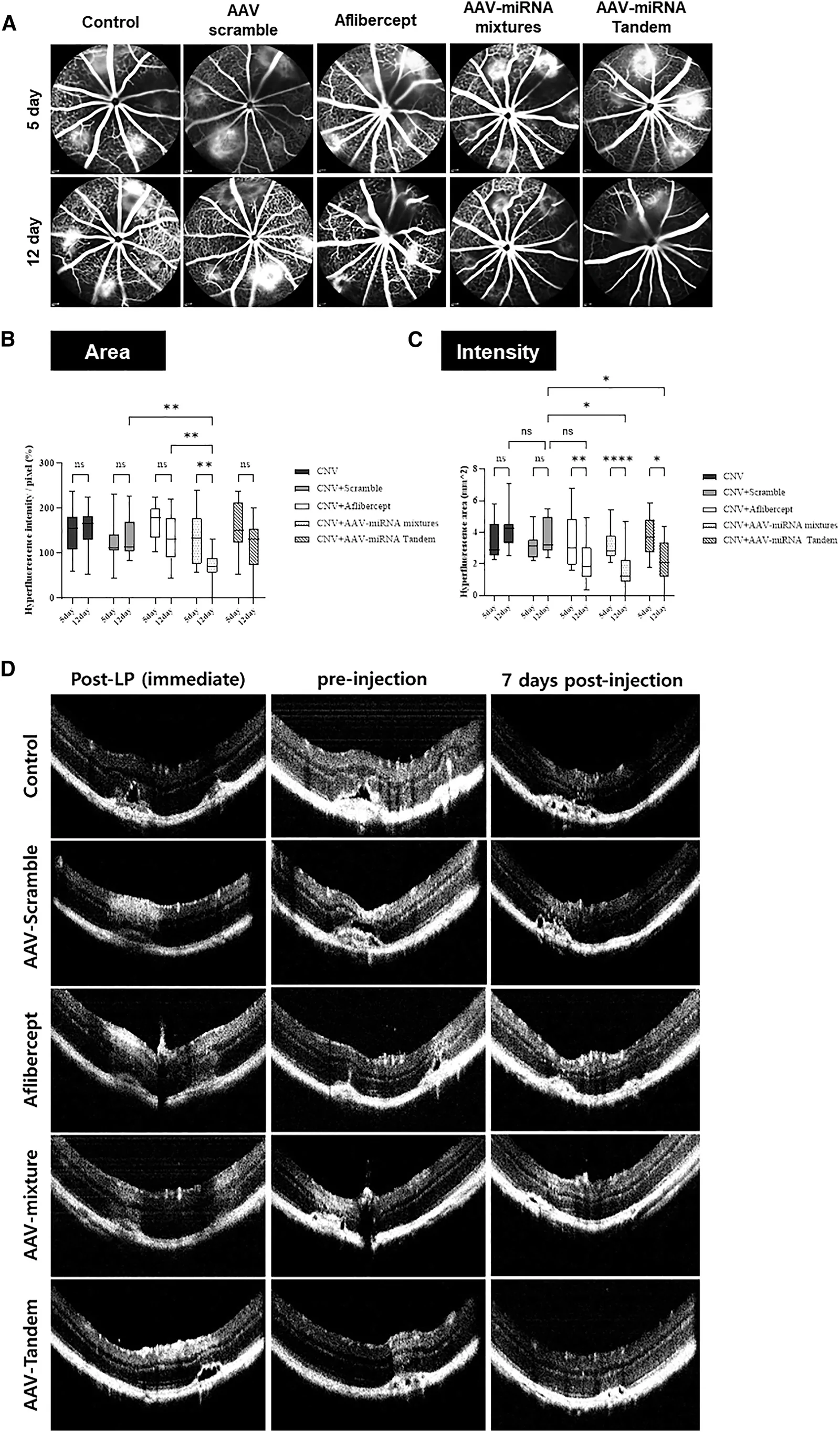
Attenuation of CNV leakage and structural lesions following rAAV2.7m8-MAPK-miR treatment assessed by FFA and OCT (A) Representative FFA images depicting CNV lesions in the control (untreated CNV), rAAV-scramble, aflibercept, rAAV2.7m8-MAPK-miR mixture, and rAAV2.7m8-MAPK-miR tandem-treated groups at 5 and 12 days post-laser photocoagulation. Clear and defined hyperfluorescent areas indicating CNV lesions were observed at day 5 across all groups. By day 12, aflibercept, rAAV2.7m8-MAPK-miR mixture, and rAAV2.7m8-MAPK-miR tandem-treated groups showed notable reduction in hyperfluorescence. (B and C) Quantitative analyses of CNV lesion areas (B) and hyperfluorescence intensities (C). Significant reductions in hyperfluorescent areas were observed at day 12 compared with day 5 in aflibercept, rAAV2.7m8-MAPK-miR mixture, and rAAV2.7m8-MAPK-miR tandem-treated groups. The rAAV2.7m8-MAPK-miR mixture-treated group exhibited a statistically significant decrease in hyperfluorescent intensity. (D) Representative OCT images acquired immediately after laser photocoagulation, prior to intravitreal injection (pre-injection), and 7 days after injection. CNV-associated structural disruptions, including subretinal lesions and retinal thickening, were evident following laser induction. Treatment with rAAV2.7m8-MAPK-miR mixture and tandem resulted in marked attenuation of CNV-associated structural abnormalities compared with control and AAV-scramble groups, and appeared to confer greater structural improvement than aflibercept. Statistical significance was analyzed using one-way ANOVA with post hoc comparisons (∗*p* < 0.05, ∗∗*p* < 0.01, ∗∗∗*p* < 0.001, ∗∗∗∗*p* < 0.0001; ns, not significant). Data are presented as mean ± SEM.

To further assess structural changes, OCT imaging was performed at multiple time points, including immediately after laser photocoagulation, prior to treatment, and 7 days post-injection (Figure 4D). Robust CNV-associated structural disruptions, including subretinal lesions and retinal thickening, were clearly observed following laser photocoagulation, confirming successful CNV induction. Notably, when comparing pre- and post-treatment OCT images, both the rAAV2.7m8-MAPK-miR mixture- and tandem-treated groups exhibited marked reductions in CNV-associated structural abnormalities. In contrast, these lesions persisted in the control and rAAV-scramble groups. These findings indicate that rAAV2.7m8-MAPK-miR treatment not only reduces vascular leakage but also alleviates CNV-related structural changes in the retina.

### The anti-angiogenic effect of rAAV2.7m8-MAPK-miR

We evaluated the anti-angiogenic effects of rAAV2.7m8-MAPK-miR by immunostaining choroidal flat-mounted CNV lesions with isolectin B4, an endothelial cell marker, and compared the results with those of the aflibercept-treated group. Mice treated with aflibercept, rAAV2.7m8-MAPK-miR mixture, and rAAV2.7m8-MAPK-miR tandem exhibited significantly reduced CNV areas compared with the untreated and rAAV-scramble-treated groups (*p* < 0.01, *p* < 0.0001, and *p* < 0.0001, respectively) (Figure 5A). No significant difference was observed between the rAAV2.7m8-MAPK-miR mixture and rAAV2.7m8-MAPK-miR tandem groups; however, both treatments resulted in a significantly greater reduction in CNV area compared with aflibercept treatment (*p* < 0.01 and *p* < 0.05, respectively) (Figure 5B).

**Figure 5:**
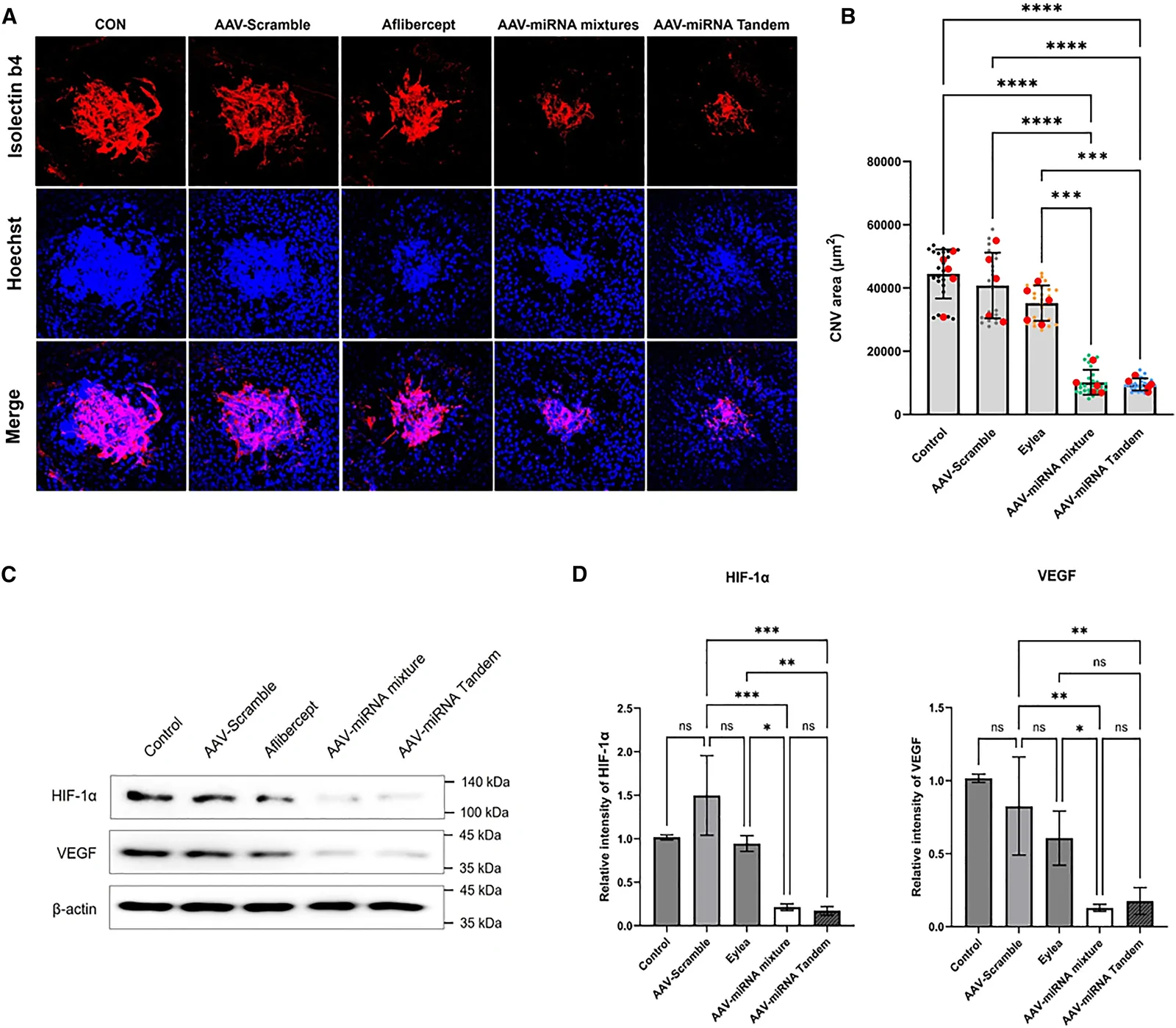
Anti-angiogenic effects of rAAV2.7M8-MAPK-miR in laser-induced CNV (A) Representative confocal images of whole-mount CNV lesions stained with isolectin B4 (red) and Hoechst nuclear staining (blue) from control (CON), rAAV-scramble, aflibercept, rAAV2.7m8-miRNA mixtures, and rAAV2.7m8-miRNA tandem treatment groups. (B) Quantification of CNV area based on isolectin B4-stained choroidal flat mounts. Five laser burns were initially generated per eye, and lesions that did not meet the predefined inclusion criteria were excluded from the analysis. Small colored dots represent all individual CNV lesions included in the analysis, whereas larger red dots represent the mean CNV area of the eligible lesions from each individual eye. Multiple CNV lesions within the same eye were considered subsamples rather than independent biological replicates; therefore, the mean CNV area per eye was used as the experimental unit for statistical analysis (*n* = 5 eyes per group). Bars represent the mean ± SEM of the eye-level means. Statistical comparisons were performed using one-way ANOVA followed by Tukey’s multiple comparisons test. ∗∗∗*p* < 0.001. (C) Representative Western blot images showing protein expression levels of HIF-1α and VEGF in retinal tissues from each experimental group, with β-actin used as a loading control. (D and E) Quantitative analysis of relative HIF-1α (D) and VEGF (E) protein expression levels normalized to β-actin. Both HIF-1α and VEGF expression were significantly reduced in the rAAV2.7m8-miRNA mixture and tandem-treated groups compared with control and rAAV-scramble groups, indicating suppression of hypoxia-driven angiogenic signaling. Statistical significance was analyzed using one-way ANOVA with post hoc comparisons (∗*p* < 0.05, ∗∗*p* < 0.01, ∗∗∗*p* < 0.001, ∗∗∗∗*p* < 0.0001; ns, not significant). Scale bars, 50 μm. Data are presented as mean ± SEM.

To further investigate whether rAAV2.7m8-MAPK-miR treatment modulates angiogenic signaling pathways downstream of MAPK activation, we evaluated the expression levels of HIF-1α and VEGF in retinal tissues by western blot analysis (Figures 5C and 5D). In the control and AAV-scramble groups, HIF-1α expression was elevated, indicating activation of hypoxia-related signaling under CNV conditions. In contrast, treatment with rAAV2.7m8-MAPK-miR mixture and tandem significantly reduced HIF-1α expression levels (*p* < 0.01–0.001). Consistent with these findings, VEGF expression was also significantly decreased in the rAAV2.7m8-MAPK-miR mixture and tandem groups compared with control and AAV-scramble groups (*p* < 0.05–0.01). These results suggest that rAAV2.7m8-MAPK-miR treatment effectively suppresses hypoxia-driven angiogenic signaling by downregulating HIF-1α and its downstream target VEGF, providing a mechanistic link between MAPK inhibition and reduced CNV progression.

### Restoration of RPE morphology by rAAV-MAPK-miR

To assess changes in RPE cell structure following rAAV2.7m8-MAPK-miR treatment, we examined the expression patterns of β-catenin, a key component of the cell-to-cell junction system, in whole-mount views. Immunostaining for β-catenin clearly delineated individual RPE cells, as shown in representative confocal images. These images were further processed into extracted outlines through manual tracing.

Our analysis revealed that the morphology of RPE cells was severely disrupted in the laser-photocoagulated area, showing irregular cellular boundaries, loss of the characteristic hexagonal shape, and disorganization of the monolayer structure. However, these morphological alterations were significantly restored by aflibercept, rAAV2.7m8-MAPK-miR mixture, and rAAV2.7m8-MAPK-miR tandem. Cell boundaries became sharper with re-emergence of continuous β-catenin staining, cell area variability decreased, and the hexagonal tiling pattern gradually re-formed, whereas rAAV-scramble failed to induce similar effects in representative confocal images (Figure 6A). To further quantify these structural changes, Voronoi diagrams were generated from the extracted images using a computational algorithm (Figures 6B and 6C). Virtual circles with a diameter of 200 μm were placed at the center of the laser-photocoagulated areas, and the area and circularity of RPE cells within these regions were measured. Treatment with aflibercept, rAAV2.7m8-MAPK-miR tandem, and rAAV2.7m8-MAPK-miR mixture significantly mitigated these morphological abnormalities, as evidenced by a reduction in cell area (Figure 6D) and an increase in cell circularity (Figure 6E). Notably, the rAAV2.7m8-MAPK-miR mixture demonstrated a significantly greater improvement in both parameters compared with aflibercept and the rAAV2.7m8-MAPK-miR tandem, indicating superior efficacy in restoring RPE morphology.

**Figure 6:**
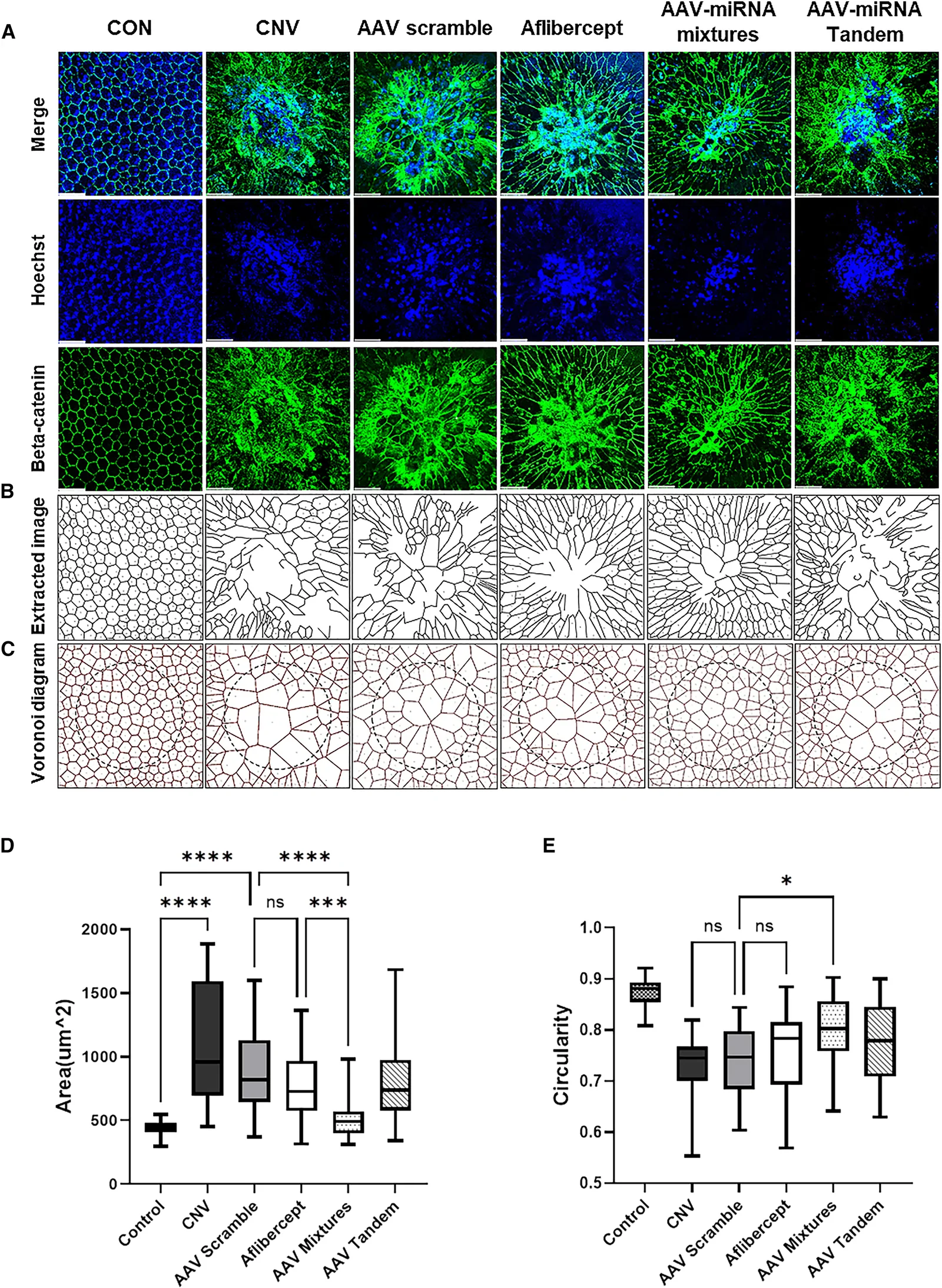
Restoration of RPE cell morphology by rAAV2.7m8-MAPK-miR treatment in laser-induced CNV (A) Representative confocal images of whole-mount immunostaining for β-catenin (green) and Hoechst nuclear staining (blue) illustrating morphological alterations in retinal pigment epithelium (RPE) cells following laser photocoagulation and subsequent treatments. “Extracted images” were generated by manually tracing β-catenin-stained outlines of individual RPE cells. Voronoi diagrams, computationally derived from the extracted images, illustrate the spatial distribution and morphology of RPE cells within the laser photocoagulation zones. (B and C) Quantitative analyses of morphological features of RPE cells using Voronoi diagrams. Virtual circles (diameter: 200 μm) were placed at the center of the laser-photocoagulated areas to measure (D) cell area (μm^2^) and (E) circularity. Significant morphological restoration was observed in the aflibercept-, rAAV2.7m8-MAPK-miR mixture-, and rAAV2.7m8-MAPK-miR tandem-treated groups compared with untreated CNV and rAAV-scramble-treated groups. Statistical significance was analyzed using one-way ANOVA with post hoc comparisons (∗*p* < 0.05, ∗∗∗*p* < 0.001, ∗∗∗∗*p* < 0.0001; ns, not significant). Scale bars, 50 μm. Data are presented as mean ± SEM.

### Suppression of epithelial-mesenchymal transition and fibrosis in CNV lesions by rAAV2.7m8-MAPK-miR

We evaluated whether rAAV2.7m8-MAPK-miR could inhibit epithelial-mesenchymal transition (EMT) and subretinal fibrosis in CNV lesions 7 days after laser photocoagulation. Flat mounts of the RPE/choroid complex were stained with antibodies against α-SMA and fibronectin, key markers of EMT. Expression levels of these marker proteins were markedly increased following laser photocoagulation, indicating that RPE cells underwent EMT in response to injury.

This EMT-related deformation was significantly inhibited by aflibercept, rAAV2.7m8-MAPK-miR mixture, and rAAV2.7m8-MAPK-miR tandem, but not by rAAV-scramble. Notably, rAAV2.7m8-MAPK-miR mixture significantly decreased α-SMA expression compared with both rAAV2.7m8-MAPK-miR tandem and aflibercept (Figures 7A and 7B). rAAV2.7m8-MAPK-miR mixture and rAAV2.7m8-MAPK-miR tandem reduced fibronectin expression to a greater extent than aflibercept (Figures 7C and 7D).

**Figure 7:**
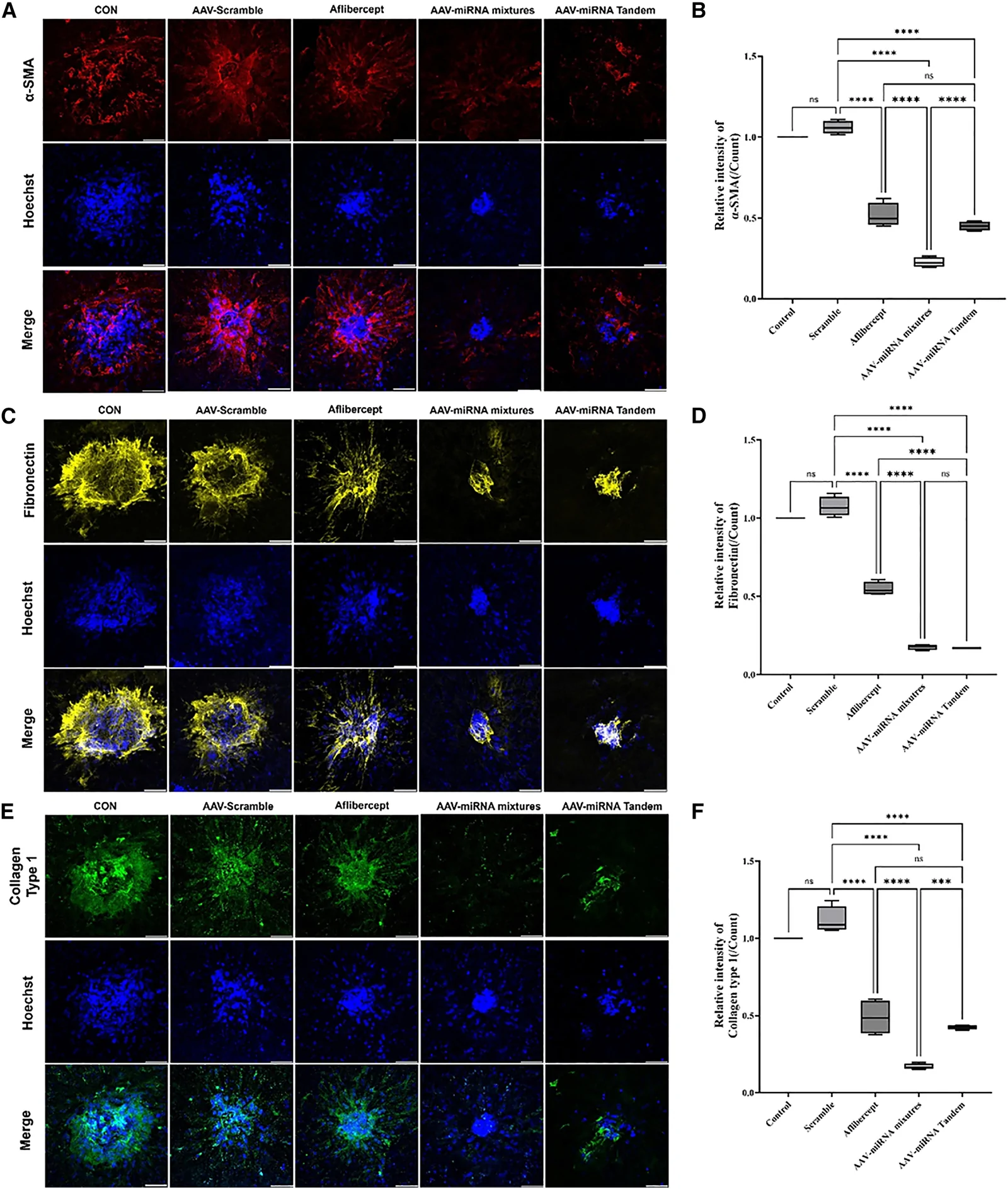
Suppression of EMT and Subretinal Fibrosis by rAAV2.7m8-MAPK-miR in CNV Lesions (A, C, E) Representative immunofluorescence images showing expression of EMT and fibrosis markers in laser-induced CNV lesions of mice treated with rAAV-scramble, aflibercept, rAAV2.7m8-MAPK-miR mixtures, and rAAV2.7m8-MAPK-miR tandem. (A) Immunostaining for α-SMA (red), (C) Fibronectin (yellow), and (E) Collagen Type I (green), with Hoechst nuclear staining (blue). (B, D, F) Quantitative analysis of immunostaining intensity, normalized to cell count, indicating the relative expression levels of (B) α-SMA, (D) Fibronectin, and (F) Collagen Type I. Both rAAV2.7m8-MAPK-miR mixtures and rAAV2.7m8-MAPK-miR tandem significantly reduced expression levels of EMT and fibrosis markers compared with untreated control and rAAV-scramble groups. Specifically, rAAV2.7m8-MAPK-miR mixtures showed a more pronounced reduction in α-SMA expression compared with aflibercept and rAAV2.7m8-MAPK-miR tandem. Statistical significance was analyzed using one-way ANOVA with post hoc comparisons (∗∗∗*p* < 0.001, ∗∗∗∗*p* < 0.0001; ns, not significant). Scale bars, 50 μm. Data are presented as mean ± SEM.

Next, we examined the anti-fibrotic effects of rAAV2.7m8-MAPK-miR on CNV lesions. Type I collagen staining of flat mounts revealed that subretinal fibrosis was significantly reduced in the aflibercept-, rAAV2.7m8-MAPK-miR mixture-, and rAAV2.7m8-MAPK-miR tandem-treated groups compared with rAAV-scramble 7 days after laser photocoagulation (Figures 7E and 7F). Notably, among these treatments, the rAAV2.7m8-MAPK-miR mixture produced the most pronounced and statistically significant reduction in subretinal fibrosis, outperforming both aflibercept and the rAAV2.7m8-MAPK-miR tandem. These results indicate that the miRNA mixture confers superior anti-fibrotic efficacy in suppressing CNV-associated subretinal fibrosis.

### Transcriptomic and functional safety evaluation of rAAV2.7m8-MAPK-miR treatment

To evaluate the safety of intravitreal rAAV2.7m8-MAPK-miR mixture delivery, full-field scotopic electroretinography (ERG) was performed in wild-type mice. Representative ERG traces demonstrated well-preserved waveform morphology in rAAV2.7m8-MAPK-miR mixture-treated eyes, comparable with those observed in control eyes across a wide range of stimulus intensities (Figure 8A). Quantitative analysis showed that scotopic a-wave amplitudes, reflecting photoreceptor function, increased with stimulus intensity in both groups and did not differ significantly between control and treated mice at any intensity tested (Figure 8B). Similarly, scotopic b-wave amplitudes, representing post-photoreceptor inner retinal activity, exhibited comparable responses between the two groups across all stimulation levels (Figure 8C). These findings indicate that intravitreal administration of rAAV2.7m8-MAPK-miR mixture does not induce detectable functional impairment of photoreceptors or inner retinal neurons in normal mice.

**Figure 8:**
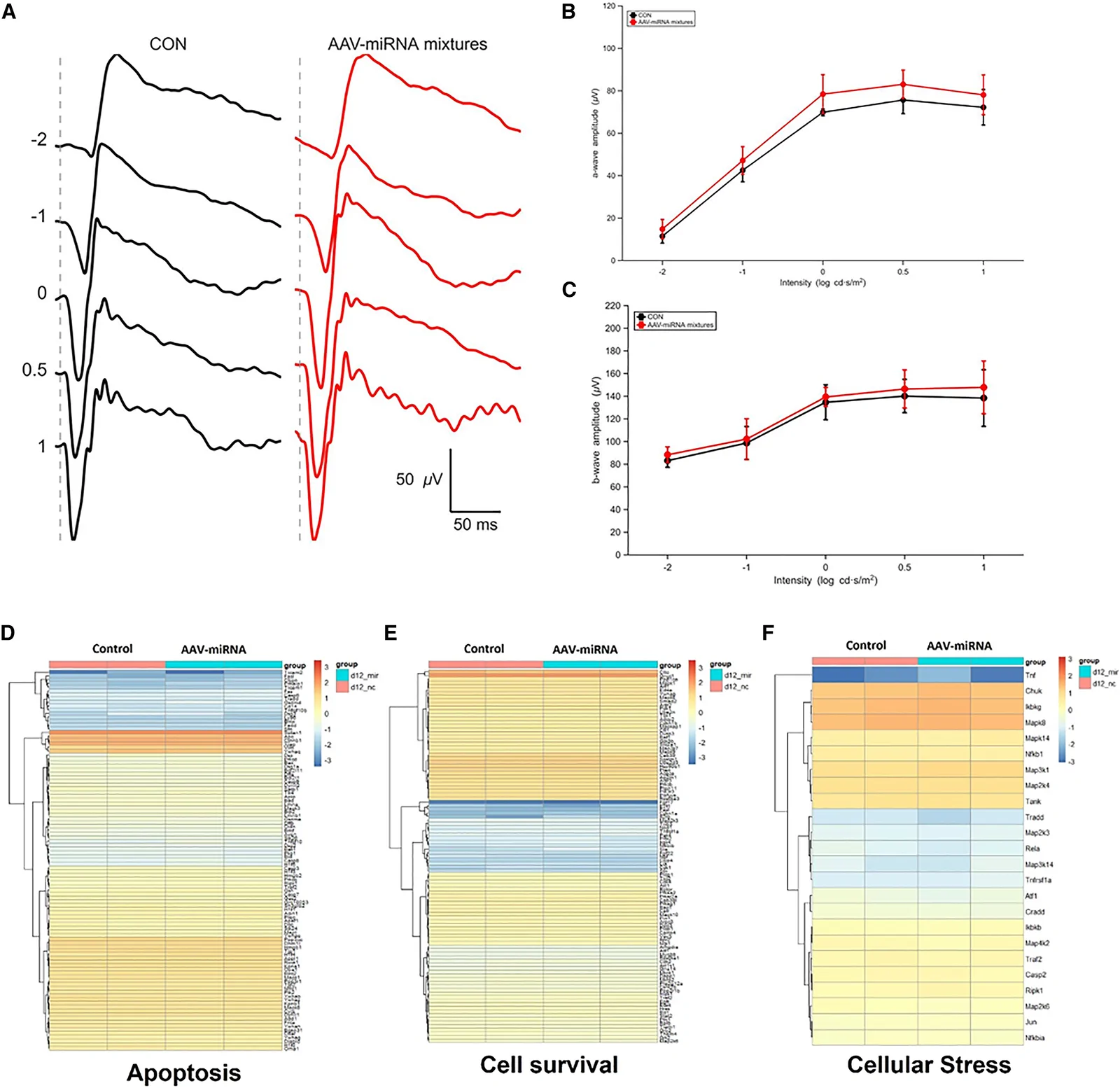
Functional and transcriptomic safety evaluation of rAAV2.7m8-MAPK-miR treatment (A) Representative full-field scotopic electroretinography (ERG) traces recorded from control and rAAV2.7m8-MAPK-miR mixture-treated eyes across a range of stimulus intensities. (B, C) Quantitative analysis of ERG responses. (B) Scotopic a-wave amplitudes and (C) b-wave amplitudes plotted as a function of stimulus intensity. No significant differences were observed between control and rAAV2.7m8-MAPK-miR-treated groups, indicating preserved photoreceptor and inner retinal function following treatment. (D–F) Transcriptomic analysis of retinal tissues from control and rAAV2.7m8-MAPK-miR-treated mice. Heatmaps showing gene expression profiles of pathways related to apoptosis (D), cell survival (PI3K/AKT signaling) (E), and cellular stress (F). Across all pathways, gene expression patterns were largely comparable between the two groups, with no distinct tandeming or consistent transcriptional alterations observed. Data are presented as mean ± SEM. Statistical analysis for ERG data was performed using two-way ANOVA with appropriate post hoc tests.

To further assess molecular safety, we performed transcriptomic analysis using RNA sequencing of retinal tissues from treated and control mice. Pathway-focused analysis revealed no significant differences in gene expression profiles between the two groups across key biological processes, including apoptosis, cell survival, and cellular stress response (Figure 8D). Heatmap analysis confirmed highly similar expression patterns, with no distinct tandeming or consistent shifts in gene expression. Notably, the majority of genes within these pathways exhibited non-significant changes, indicating the absence of detectable transcriptomic perturbation. Collectively, these results demonstrate that intravitreal delivery of rAAV2.7m8-MAPK-miR mixture does not induce functional or molecular alterations in the normal retina, supporting the overall safety of this multi-miRNA-based AAV therapeutic approach.

## Discussion

Emerging evidence suggests that the MAPK signaling pathway plays a crucial role in the pathogenesis and progression of AMD.^34^^,^^35^^,^^36^ Oxidative stress, a key driver of AMD, activates MAPK components—including ERK, JNK, and p38—which contribute to retinal pigment epithelium (RPE) cell apoptosis, inflammatory responses, and pathological angiogenesis.^16^^,^^17^^,^^37^^,^^38^ Aberrant MAPK activation has been shown to exacerbate RPE degeneration and promote CNV, highlighting its significance as a molecular target for AMD therapy. However, direct inhibition of MAPK signaling has posed therapeutic challenges due to associated adverse effects,^39^^,^^40^ including retinal toxicity, increased risk of retinal fibrosis, photoreceptor degeneration, and impaired retinal pigment epithelium (RPE) function, necessitating the development of alternative therapeutic strategies. One promising approach involves the use of AAV vectors to deliver miRNAs that selectively regulate MAPK pathway components. By leveraging AAV-mediated gene transfer, miRNA-based therapy offers a more precise and sustained modulation of MAPK signaling, potentially overcoming the limitations of conventional pharmacological inhibitors while minimizing off-target effects. This strategy holds significant potential for AMD treatment by effectively targeting MAPK-driven pathological processes while improving safety and therapeutic efficacy.

To evaluate the therapeutic potential of MAPK-targeting AAV, we utilized ARPE19 cells and a laser-induced CNV mouse model, while acknowledging their inherent limitations. ARPE19 cells do not fully recapitulate the physiological properties of native RPE, and the laser-induced CNV model represents an acute injury model rather than the chronic pathology of human AMD. We evaluated three miRNAs—hsa-miR-122-5p, hsa-let-7a-5p, and hsa-miR-26a-5p—that collectively target key components of the MAPK signaling pathway. These miRNAs were selected based on their strong association with all MAPK-related genes and their ability to modulate oxidative stress in RPE cells. Notably, these miRNAs are highly conserved between human and mouse, with identical sequences for miR-122-5p and let-7a-5p and only a minor variation in miR-26a-5p outside the seed region, supporting the translational relevance of our findings. Each miRNA plays a distinct role in regulating MAPK pathway activity and contributes to angiogenesis, EMT and subretinal fibrosis suppression.

hsa-miR-122-5p, widely studied in hepatic metabolism and inflammation,^41^ has recently been implicated in retinal pathophysiology.^30^ Previous studies have reported that miR-122-5p can suppress pro-inflammatory cytokine production and oxidative stress by targeting MAPK pathway components, including ERK and JNK.^30^ Similarly, hsa-let-7a-5p is a well-conserved miRNA involved in cellular differentiation and stress response.^27^^,^^29^ It has been reported to inhibit EMT and reduce fibrosis, both of which are crucial factors in the progression of AMD. hsa-miR-26a-5p is a well-documented tumor suppressor miRNA with anti-angiogenic properties^32^^,^^42^^,^^43^ and has been shown to target components of the MAPK pathway, including p38. In the present study, treatment with the multi-miRNA formulations resulted in reduced oxidative stress-induced apoptosis, decreased expression of fibrotic markers such as α-SMA and fibronectin, and suppression of CNV and endothelial cell proliferation. While our experimental design does not allow us to distinguish the individual contribution of each miRNA, these effects are likely the result of the combined and complementary actions of miR-122-5p, let-7a-5p, and miR-26a-5p. Collectively, this multi-miRNA approach may enable broader modulation of MAPK-associated pathological processes, including inflammation, angiogenesis, fibrosis, and oxidative stress. Therefore, delivering these miRNAs together represents a promising therapeutic strategy for effectively treating AMD by simultaneously targeting key disease-driving pathways.

We observed that while both the rAAV2.7m8-MAPK-miR mixture and the rAAV2.7m8-MAPK-miR tandem effectively suppressed CNV, the tandem formulation exhibited relatively limited efficacy in inhibiting EMT and subretinal fibrosis. One possible explanation for this discrepancy is the lower miRNA expression levels observed in the tandem formulation, as demonstrated by quantitative PCR. In addition, differences in miRNA processing efficiency or intracellular handling when multiple miRNAs are co-expressed from a single vector may contribute to this effect, although this was not directly investigated in the present study. Consistent with this possibility, when multiple miRNAs are packaged within a single construct, they may compete for processing by the cellular miRNA biogenesis machinery, leading to reduced efficiency in individual miRNA maturation and function.^44^^,^^45^ To address this issue, we incorporated separate promoters for each miRNA within the rAAV2.7m8-MAPK-miR tandem to enable independent transcription and reduce potential transcriptional interference between expression cassettes, although post-transcriptional competition may still occur. Despite this design, the therapeutic effects of the tandem formulation on EMT and fibrosis remained less pronounced compared with the mixture formulation. This suggests that factors beyond transcriptional competition—such as saturation of the RNA-induced silencing complex (RISC) or altered miRNA stability—may contribute to the observed differences in efficacy.^46^ Future optimization of tandem design will be essential to further improve therapeutic outcomes.

Nevertheless, the rAAV2.7m8-MAPK-miR tandem offers certain advantages, particularly in terms of delivery efficiency and vector production. By packaging multiple miRNAs into a single vector, the tandem formulation allows for the simultaneous delivery of multiple therapeutic miRNAs, potentially reducing the required viral dose and minimizing the risk of immune responses. Additionally, using a single vector simplifies large-scale production and regulatory approval processes compared with delivering multiple separate vectors. These advantages make the rAAV2.7m8-MAPK-miR tandem a promising approach, provided that future optimizations can overcome the current limitations in miRNA processing and efficacy. Future improvements in multi-miRNA delivery systems may benefit from alternative expression architectures beyond tandem Pol-III cassettes, which can be associated with promoter interference and imbalanced expression. In the present study, we did not systematically compare different vector configurations or promoter systems, representing a limitation that should be addressed in future work. For example, Pol-II-driven miRNA tandems have been widely used to express multiple miRNAs from a single primary transcript that is subsequently processed by the endogenous miRNA biogenesis machinery.^47^ This strategy enables coordinated expression of multiple miRNAs while reducing promoter interference observed in multi-cassette Pol-III designs. More recently, artificial miRNA concatemers expressed under tissue-specific Pol-II promoters have been developed for retinal gene therapy applications, allowing controlled and cell-type-restricted expression of multiple therapeutic miRNAs from a single transcript.^48^ Such approaches provide improved transcriptional balance and reduced vector complexity compared with multi-cassette designs. Together, these emerging strategies represent promising alternatives for improving the stability and therapeutic efficacy of multi-miRNA delivery systems for AMD.

The findings of this study provide compelling evidence that rAAV2.7m8-mediated delivery of MAPK-targeting miRNAs represents a promising therapeutic approach for AMD. The ability of rAAV2.7m8-MAPK-miR to effectively reduce CNV leakage, inhibit angiogenesis, and mitigate EMT and subretinal fibrosis suggests that this strategy could serve as an alternative or adjunct to anti-VEGF therapy. Importantly, the long-term expression potential of AAV vectors may alleviate the need for frequent intravitreal injections, thereby improving patient compliance and reducing treatment burden. Although the present study primarily focused on the short-term therapeutic efficacy of a single intravitreal administration of rAAV2.7m8-MAPK-miR, we acknowledge that long-term treatment outcomes and dose-dependent responses were not fully explored. This represents an important limitation, as sustained therapeutic efficacy and durability are critical considerations for clinical translation. In the current study, short-term safety evaluations revealed no detectable adverse effects on retinal function following intravitreal delivery, supporting the initial tolerability of this approach. However, comprehensive long-term investigations are warranted to assess the durability of miRNA expression, the sustainability of therapeutic effects, and the potential for delayed toxicity or immunogenicity associated with rAAV2.7m8-mediated miRNA delivery. Future studies should therefore focus on optimizing dosing strategies, including evaluation of different vector doses and repeated or staged administration regimens, to determine the minimal effective dose and long-term efficacy profile. In parallel, optimization of delivery routes may further enhance therapeutic outcomes while minimizing potential adverse effects. Additionally, validation in larger animal models with longer follow-up periods, followed by carefully designed clinical studies, will be essential to establish the translational feasibility and long-term safety of rAAV-based multi-miRNA therapy for neovascular AMD.

In conclusion, this study demonstrates that rAAV2.7m8-mediated delivery of MAPK-targeting miRNAs effectively suppresses CNV progression and restores RPE structure in an AMD model. Notably, this study demonstrates that the delivery of three MAPK-targeting miRNAs using rAAV vectors represents a promising miRNA-based gene therapy approach for AMD. The superior efficacy of the rAAV2.7m8-MAPK-miR mixture over the rAAV2.7m8-MAPK-miR tandem underscores the importance of individual miRNA regulation in achieving optimal therapeutic outcomes. These findings not only validate the therapeutic potential of miRNA-based interventions but also pave the way for further development and clinical application of durable gene therapy strategies for AMD.

## Materials and methods

### Preparation of mixture of three rAAV2.7m8-miRNAs and intravitreal injection

The design and production of rAAV2.7m8-MAPK-miR mixture and rAAV2.7m8-MAPK-miR tandem were carried out by VectorBuilder. All vector concentrations were greater than 10^12^ genome copies/mL. Five days after laser photocoagulation and FFA, rAAV2.7m8-MAPK-miR mixture or rAAV-scramble was intravitreally injected into one eye at a total dose of 1 × 10^9^ genome copies (GC) per eye under anesthesia. For the miRNA mixture, the total dose consisted of equal amounts of the three individual AAV vectors, with each vector administered at approximately 0.33 × 10^9^ GC per eye. A puncture site was carefully created approximately 2 mm posterior to the limbus using a 30-G beveled needle under an operating microscope. The rAAV was injected through the puncture site using a NanoFil syringe with a 34-G beveled needle (World Precision Instruments Inc., Sarasota, FL, USA). Aflibercept was injected at a concentration of 40 μg/μL per eye.

### Cells and cell culture

ARPE-19 cells were purchased from the American Type Culture Collection (ATCC, Manassas, VA, CRL-2302) and cultured in DMEM/F-12 from Welgene (Gyeongsan, Korea, LM002-01) with 10% fetal bovine serum (HyClone, Logan, UT, SH30071) and 1% penicillin-streptomycin (Gibco, Gaithersburg, MD, 15140-122) at 37°C and 5% CO_2_. At 80%–90% cell confluency, cells were transduced with rAAV-MAPK-miR mixture or rAAV-MAPK-miR tandem at a total dose of 1 × 10^9^ GC/mL for 5 days. For the miRNA mixture, equal amounts of each individual AAV vector were applied. For the H_2_O_2_ treatment groups, the cells were exposed to 200 μM H_2_O_2_ (Sigma-Aldrich, 386790-M) for 2 h.

### Animals

All animal experiments were conducted in strict compliance with the “Guide for the Care and Use of Laboratory Animals” and the “Association for Research in Vision and Ophthalmology Statement for the Use of Animals in Ophthalmic and Vision Research.” These experiments received approval from the Institutional Animal Care and Use Committee of Soonchunhyang University Hospital, Bucheon.

C57BL/6J mice were obtained from Orient Bio (Seongnam, South Korea) and DBL Inc. (Eumseong, South Korea), both of which are authorized distributors of Jackson Laboratory mouse strains in Korea. 8-week-old male C57BL/6J mice were used in this study. The mice were housed in a controlled environment with regulated temperature and humidity, following a 12/12-h light/dark cycle. They had unrestricted access to food and water.

To facilitate anesthesia, we administered an intraperitoneal injection of a combination of Zoletil 50 (Virbac; Carros Cedex, France) and Rompun (Bayer Healthcare, Leverkusen, Germany) at a weight ratio of 4:1. Prior to all laser treatments and examinations, pupil dilation was carried out using a mixture of 0.5% tropicamide and 0.5% phenylephrine (both w/v), known as Tropherine (Hanmi Pharm, Seoul, South Korea).

### Laser-induced CNV in mice

Laser photocoagulation was performed following the previously described method.^49^ Photocoagulation involved the use of a diode ophthalmic laser system (Neodymium-doped yttrium aluminum garnet [Nd:YAG], 532 nm; Topcon Medical Laser Systems, Livermore, CA, USA). This procedure was conducted with the mice under anesthesia and their pupils dilated.

The photocoagulation spots were created with a 200-μm spot diameter, a 20 ms duration, and a power of 120 mW. Four or five laser burns were applied per eye and positioned at the 12, 2, 5, 7, and 10 o’clock locations around the optic nerve, with the laser focused on the retinal pigmented epithelium. The appearance of a vaporization bubble was used as an indicator of successful Bruch’s membrane rupture. Five mice were included in each experimental group, and only one eye per animal was used for analysis. Lesions were excluded from quantitative analysis if they exhibited obvious hemorrhage, inadequate rupture of Bruch’s membrane (absence of bubble formation), severe ocular damage, infection, or poor image quality. Animals showing signs of ocular damage or infection after laser treatment were also excluded. More than 80% of induced lesions met the predefined inclusion criteria and were included in the final CNV analysis.

### Experimental design

All mice were assigned to five groups based on the day of sacrifice after CNV induction: (1) CNV12d, (2) CNV 5d+ AAV-miRNAs scramble 7d, (3) CNV 5d+ Afilbercept 7d, (4) CNV 5d+ rAAV-MAPK-miR mixture 7d, and (5) CNV 5d+ rAAV-MAPK-miR tandem 7d.

Five days after LP, CNV formation was confirmed in all mice through FFA imaging. Immediately afterward, rAAV-scramble, rAAV-MAPK-miR mixture, and rAAV-MAPK-miR tandem were administered via intravitreal injections to unilateral eye of all groups, except for the CNV 12d group. On the 12th day after LP treatment, following FFA re-imaging to confirm the therapeutic effects, all experimental animals were sacrificed according to the experimental analysis methods.

### TaqMan probe PCR

Quantitative real-time PCR (qPCR) analysis of miRNAs was performed using the TaqMan MicroRNA Assay Kit (Applied Biosystems, Foster City, CA, USA, 4427975) according to the manufacturer’s protocol. Briefly, total RNA, including miRNA, was extracted from cell culture media samples using the miRNeasy Serum/Plasma Kit (Qiagen, Hilden, Germany, 217184). RNA samples were then reverse-transcribed using TaqMan MicroRNA Reverse Transcription Kit and specific RT primers for hsa-miR-122-5p, hsa-let-7a-5p, and hsa-miR-26a-5p. qPCR reactions were conducted in triplicate using TaqMan Universal PCR Master Mix and specific TaqMan probes on a QuantStudio 5 Real-Time PCR System (Applied Biosystems). miRNA expression levels were normalized to to no template control to obtain ΔCt values, and relative quantification was calculated using the 2ˆ−ΔΔCt method.

### Western blot analysis

The expression levels of ERK, JNK, p38, p-p38, p-JNK, and p-ERK (1:1,000; Cell Signaling Technology, 9102, 9252, 8690, 4511S, 9255S, 9101S) along with β-actin (1:1,000; Santa Cruz, sc-47778) in the retina and RPE-choroid complex were analyzed by Western blotting.

Mice were sacrificed in a CO_2_ chamber under deep anesthesia, and their eyeballs were immersed in PBS to remove connective tissues, cornea, and optic nerve. After separating the retina and RPE/choroid complex from the eyecup, samples from both eyes were pooled and used for the experiment. For protein extraction, tissues were homogenized and treated with RIPA II lysis buffer (GenDEPOT, Barker, TX, USA, R4200-010) containing Xpert phosphatase inhibitor cocktail and Xpert protease inhibitor cocktail (GenDEPOT, TX, USA) on ice for 30 min. Following centrifugation at 13,000 g, 4°C, for 15 min, the supernatant containing protein lysate was collected. Protein concentration was determined using the Pierce BCA Protein Assay kit (Thermo Scientific, Middlesex, MA, USA, 23227) according to the manufacturer’s instructions. The protein lysate was then dissolved at 1 μg/μL in 5X protein sample buffer (Elpis Biotech, Daejeon, Korea, EBA-1052) and boiled for 10 min at 95°C. Samples were loaded onto sodium dodecyl sulfate-polyacrylamide gel electrophoresis gels and transferred to polyvinylidene difluoride membranes (Merck, Kenilworth, NJ, USA, IPVH08100).

Subsequently, membranes were blocked with 5% (w/v) skim milk in PBS and incubated on a shaker at room temperature for 1 h. Primary antibodies were incubated overnight at 4°C on a shaker. The next day, after washing, membranes were exposed to secondary antibodies conjugated with horseradish peroxidase (GenDEPOT, TX, USA) at room temperature for 2 h. Protein bands were visualized using EzWestLumi plus (ATTO Co., Tokyo, Japan, 2332638) and imaged with the Azure Biosystems C280 digital system (Azure Biosystems, Dublin, CA, USA). Blot bands were quantified using ImageJ software (National Institutes of Health, MD, USA).

### Fundus fluorescent angiography

FFA was conducted in accordance with previous descriptions, with the mice placed under anesthesia and their pupils dilated. We utilized a confocal, scanning laser ophthalmoscope (Heidelberg Retina Angiograph 2; Heidelberg Engineering, Heidelberg, Germany) to assess the status of CNV. FFA images were taken 3 to 5 min following the intraperitoneal injection of 150 μL of 1% (w/v) fluorescein sodium (Fluorescite; Akorn, Lake Forest, IL, USA). FFA images captured at the same time points were subsequently analyzed using ImageJ software (National Institutes of Health, Bethesda, MD, USA). To do this, the maximal leakage areas were manually selected, and these areas were quantified in pixels and converted to μm^2^ utilizing the “Scale” tool.

### OCT

*In vivo* retinal imaging was performed using spectral-domain OCT (SD-OCT) to evaluate structural changes associated with laser-induced CNV. A preclinical retinal SD-OCT system developed by the Korea Research Institute of Standards and Science (KRISS) was used for imaging. OCT imaging was conducted immediately after laser photocoagulation (Post-LP), prior to intravitreal injection (pre-injection), and 7 days after treatment. Cross-sectional B-scan images centered on laser-induced lesions were acquired for each eye. CNV-associated structural alterations, including subretinal hyperreflective material, retinal thickening, and disruption of the outer retinal layers, were assessed.

### Immunofluorescence analysis

Prior to sacrifice, mice were deeply anesthetized and perfused through the heart with 0.1 M phosphate buffer (PB) containing 1,000 U/mL heparin, followed by perfusion with 4% (v/v) paraformaldehyde (PFA) in 0.1 M PB (Biosesang, Seongnam, Korea, PC2205-100-74, PR2296-050-74). After removing the anterior segment to create eyecups from enucleated eyes, they were fixed in 4% PFA at 4°C for 1 h. The eyecups were dehydrated in 30% (w/v) sucrose overnight and embedded in a frozen section compound from Leica Biosystems (Richmond, IL, USA). Transverse tissue cryosections of 10 μm were cut and stored at −80°C until further use.

The slides were dried at 60°C for 30 min and washed with PB saline (PBS) for 15 min. Permeabilization was achieved by incubating with 0.1% (v/v) Triton X-100 in PBS for 10 min. Non-specific binding was blocked by incubating samples with 5% (v/v) normal donkey serum in PBS for 1 h at room temperature. Subsequently, primary antibodies, diluted in blocking solution were applied and incubated overnight at 4°C. The following day, slides were washed with PBS and then incubated with secondary antibodies (Alexa Fluor 488, 568 or 647, dilution 1:1,000; Invitrogen Corp., Carlsbad, CA, USA) at room temperature for 2 h. In the rAAV2.7m8-MAPK-miR mixture treatment group, Alexa Fluor 647-conjugated secondary antibodies were specifically used to avoid interference with the fluorescent signals (mCherry, TagBFP2, EGFP) emitted from each miRNA construct. Counterstaining was performed using Hoechst 33342 (Invitrogen, H1399). Finally, slides were mounted with mounting medium (Abcam, Cambridge, UK, AB104135) and imaged using a microscope equipped with a Leica confocal camera (DMI8; Leica Camera, Wetzlar, Germany). All staining procedures were conducted in a dark and humid chamber.

For RPE whole-mount preparation, the neural retina was carefully removed from the RPE-choroid complex under a dissecting microscope. The isolated RPE-choroid tissue was washed with PBS and incubated in 70% ethanol at room temperature for 30 min to enhance tissue permeability.

Subsequently, RPE whole mounts were processed using the same immunofluorescence staining protocol as described for retinal sections, including permeabilization, blocking, primary and secondary antibody incubation, nuclear counterstaining, and mounting. The stained whole mounts were flat-mounted on glass slides and imaged using a Leica DMI8 confocal microscope. All staining procedures were performed under light-protected, humidified conditions. Primary antibodies used included anti-CD31 (BD Biosciences, San Jose, CA, USA, 550274), αSMA (Sigma-Aldrich, St Louis, MO, A2547), fibronectin (Abcam, Cambridge, UK, ab2413), anti-β-catenin (Abcam, Cambridge, UK, ab22656), and anti-collagen type I (Abcam, Cambridge, UK, ab6308), anti-phospho-ERK (Cell Signaling Technology, Danvers, MA, USA, 4370), anti-phospho-JNK (Cell Signaling Technology, 4671), and anti-phospho-p38 (Cell Signaling Technology, 9211).

### Voronoi diagram

To assess the variation in morphology of RPE cells in 5 groups of mice: (1) Control; (2) AAV-miRNAs scramble; (3) Afilbercept; (4) rAAV-MAPK-miR mixture; (5) rAAV-MAPK-miR tandem, the Voronoi diagram algorithm was utilized. This was performed using Rhinoceros 5.0 software (Roberts McNeel & Associates, Seattle, WA, USA) on β-catenin-stained RPE cells. RPE cells with intact β-catenin staining from five laser-treated mice retinae were averaged for analysis. The mean area and circularity of RPE cells stained with β-catenin within 200-μm-diameter circles were estimated and calculated using ImageJ software (National Institutes of Health, MD, USA).

### ERG

Full-field ERG was performed using the Celeris System (Diagnosys LLC, Lowell, MA, USA) to assess retinal function. Mice were dark-adapted overnight (at least 12 h) prior to recording. All procedures were conducted under dim red light. ERG responses were recorded using the built-in contact lens electrode of the Celeris system, which simultaneously served as a corneal electrode and light stimulator. A reference electrode was placed subcutaneously in the forehead, and a ground electrode was positioned in the tail. Body temperature was maintained at 37°C using a heating pad throughout the experiment. Scotopic ERG responses were recorded under dark-adapted conditions using a series of flash intensities ranging from −2.0 to 1.0 log cd·s/m^2^. For each intensity, multiple responses were averaged to improve signal quality. The a-wave amplitude was measured from baseline to the trough of the negative deflection, reflecting photoreceptor function, and the b-wave amplitude was measured from the a-wave trough to the peak of the positive deflection, representing inner retinal activity.

### RNA extraction and library preparation

Total RNA was isolated using TRIzol reagent (Invitrogen). RNA integrity was assessed using an Agilent 2100 Bioanalyzer (Agilent Technologies, Amstelveen, The Netherlands), and quantification was performed with a NanoDrop ND-2000 Spectrophotometer (Thermo Fisher Scientific). Sequencing libraries were prepared using the TruSeq Stranded Total RNA Library Prep Globin Kit (Illumina, San Diego, CA, USA). mRNA was isolated using the Poly(A) RNA Selection Kit (LEXOGEN, Inc., Vienna, Austria). The isolated mRNA was subsequently used for cDNA synthesis and shearing, following the manufacturer’s instructions. Libraries were indexed using Illumina indexes 1–12 and enriched via PCR. Final library quality and mean fragment size were determined using the TapeStation HS D1000 ScreenTape (Agilent Technologies). Library quantification was conducted via qPCR using a specialized quantification kit on a StepOne Real-Time PCR System (Life Technologies, Carlsbad, CA, USA). High-throughput sequencing was performed as 100-bp paired-end reads on a NovaSeq 6000 platform (Illumina).

### Bioinformatics analysis

Quality control of raw sequencing data was performed using FastQC v0.11.9 (Babraham Institute, Cambridge, UK; https://www.bioinformatics.babraham.ac.uk/projects/fastqc/(accessed on [M40.1][HC40.2]13 March 2026)). Adapters and low-quality reads (Quality Score <20) were removed using Trimmomatic v.0.38 (https://www.usadellab.org/cms/?page=trimmomatic (accessed on 13 March 2026)).^50^ The resulting trimmed reads were mapped to the mouse reference genome (build mm10) using HISAT2.^51^ Read counts were extracted using StringTie,^52^ and differential gene expression (DEG) analysis was performed using a generalized linear model quasi-likelihood F-test within the edgeR package^53^ in R (R development Core Team; The R Foundation for Statistical Computing, Vienna, Austria). Gene expression visualization was performed using the pheatmap package in R (The R Foundation for Statistical Computing; https://CRAN.R-project.org/package=pheatmap (accessed on March 26, 2026)), with expression levels plotted without scaling.

### Statistical analysis

All experiments were independently repeated at least thrice. The statistical significance of the differences was analyzed by one-way analysis of variance (ANOVA) followed by Tukey’s post hoc test using GraphPad Prism 9 software (GraphPad Software, Inc., San Diego, CA, USA). The significant differences are indicated by a single asterisk (∗*p* < 0.05), a double asterisk (∗∗*p* < 0.01), or a triple asterisk (∗∗∗*p* < 0.001). All data are represented as mean ± SEM.

## Data and code availability

The datasets generated and/or analyzed during the present study are available from the corresponding author on reasonable request.

## Acknowledgments

This research was supported by the 10.13039/501100014188Ministry of Science and ICT of the Republic of Korea and the National Research Foundation of Korea (NRF) grant funded by the Korean government (RS-2025-16063013). It was also supported by the Bio&Medical Technology Development Program of the National Research Foundation (NRF) funded by the Korean Government (MSIT) (RS-2025-02217948). Additionally, this work was partially supported by the research fund of Soonchunhyang University.

## Author contributions

J.W.H. contributed to conducting the experiments, data acquisition, data analysis, and writing of the manuscript. J.Y.Y., Y.K., S.A., E.Y., and I.H.P. contributed to conducting the experiments, data acquisition, and data analysis. S.-W.L., H.-J.L., T.G.L., H.S.P., and J.H.K. contributed to data acquisition. J.S.C., W.H.J., and H.S.C. contributed to data analysis. T.K.P. contributed to the study design, conducting the experiments, experiment execution, data analysis, and manuscript writing. All authors reviewed and approved the final version of the manuscript.

## Declaration of interests

The authors declare no competing interests.

## Declaration of generative AI and AI-assisted technologies in the writing process

During the preparation of this work, the author(s) used ChatGPT to check some parts of the text for spelling and grammar errors. After using this tool, the author(s) reviewed and edited the content as needed and take(s) full responsibility for the content of the publication.

## Supplemental information

Document S1. Figures S1–S8 and Table S1

Document S2. Article plus supplemental information

## References

[1] Vyawahare H., Shinde P. (2022). Age-Related Macular Degeneration: Epidemiology, Pathophysiology, Diagnosis, and Treatment. Cureus.

[2] Coleman H.R., Chan C.C., Ferris F.L., Chew E.Y. (2008). Age-related macular degeneration. Lancet.

[3] Lim L.S., Mitchell P., Seddon J.M., Holz F.G., Wong T.Y. (2012). Age-related macular degeneration. Lancet.

[4] Nishijima K., Ng Y.S., Zhong L., Bradley J., Schubert W., Jo N., Akita J., Samuelsson S.J., Robinson G.S., Adamis A.P., Shima D.T. (2007). Vascular endothelial growth factor-A is a survival factor for retinal neurons and a critical neuroprotectant during the adaptive response to ischemic injury. Am. J. Pathol..

[5] Saint-Geniez M., Maharaj A.S.R., Walshe T.E., Tucker B.A., Sekiyama E., Kurihara T., Darland D.C., Young M.J., D'Amore P.A. (2008). Endogenous VEGF is required for visual function: evidence for a survival role on muller cells and photoreceptors. PLoS One.

[6] Barikian A., Mahfoud Z., Abdulaal M., Safar A., Bashshur Z.F. (2015). Induction with intravitreal bevacizumab every two weeks in the management of neovascular age-related macular degeneration. Am. J. Ophthalmol..

[7] Kaiser P.K. (2013). Emerging therapies for neovascular age-related macular degeneration: drugs in the pipeline. Ophthalmology.

[8] van Wijngaarden P., Qureshi S.H. (2008). Inhibitors of vascular endothelial growth factor (VEGF) in the management of neovascular age-related macular degeneration: a review of current practice. Clin. Exp. Optom..

[9] Krebs I., Glittenberg C., Ansari-Shahrezaei S., Hagen S., Steiner I., Binder S. (2013). Non-responders to treatment with antagonists of vascular endothelial growth factor in age-related macular degeneration. Br. J. Ophthalmol..

[10] Kuroda Y., Yamashiro K., Miyake M., Yoshikawa M., Nakanishi H., Oishi A., Tamura H., Ooto S., Tsujikawa A., Yoshimura N. (2015). Factors Associated with Recurrence of Age-Related Macular Degeneration after Anti-Vascular Endothelial Growth Factor Treatment: A Retrospective Cohort Study. Ophthalmology.

[11] Chae B., Jung J.J., Mrejen S., Gallego-Pinazo R., Yannuzzi N.A., Patel S.N., Chen C.Y., Marsiglia M., Boddu S., Freund K.B. (2015). Baseline Predictors for Good Versus Poor Visual Outcomes in the Treatment of Neovascular Age-Related Macular Degeneration With Intravitreal Anti-VEGF Therapy. Investig. Ophthalmol. Vis. Sci..

[12] Mettu P.S., Allingham M.J., Cousins S.W. (2021). Incomplete response to Anti-VEGF therapy in neovascular AMD: Exploring disease mechanisms and therapeutic opportunities. Prog. Retin. Eye Res..

[13] Maurya M., Bora K., Blomfield A.K., Pavlovich M.C., Huang S., Liu C.H., Chen J. (2023). Oxidative stress in retinal pigment epithelium degeneration: from pathogenesis to therapeutic targets in dry age-related macular degeneration. Neural Regen. Res..

[14] Wen X.Y., Yang N., Gao Y., Ma W.N., Fu Y., Geng R.F., Zhang Y.L. (2024). PRDX1 exerts a photoprotection effect by inhibiting oxidative stress and regulating MAPK signaling on retinal pigment epithelium. BMC Ophthalmol..

[15] Chan C.M., Huang J.H., Lin H.H., Chiang H.S., Chen B.H., Hong J.Y., Hung C.F. (2008). Protective effects of (-)-epigallocatechin gallate on UVA-induced damage in ARPE19 cells. Mol. Vis..

[16] Roduit R., Schorderet D.F. (2008). MAP kinase pathways in UV-induced apoptosis of retinal pigment epithelium ARPE19 cells. Apoptosis.

[17] Glotin A.L., Calipel A., Brossas J.Y., Faussat A.M., Tréton J., Mascarelli F. (2006). Sustained versus transient ERK1/2 signaling underlies the anti- and proapoptotic effects of oxidative stress in human RPE cells. Investig. Ophthalmol. Vis. Sci..

[18] Du H., Sun X., Guma M., Luo J., Ouyang H., Zhang X., Zeng J., Quach J., Nguyen D.H., Shaw P.X. (2013). JNK inhibition reduces apoptosis and neovascularization in a murine model of age-related macular degeneration. Proc. Natl. Acad. Sci. USA.

[19] Guma M., Rius J., Duong-Polk K.X., Haddad G.G., Lindsey J.D., Karin M. (2009). Genetic and pharmacological inhibition of JNK ameliorates hypoxia-induced retinopathy through interference with VEGF expression. Proc. Natl. Acad. Sci. USA.

[20] Broughton J.P., Lovci M.T., Huang J.L., Yeo G.W., Pasquinelli A.E. (2016). Pairing beyond the Seed Supports MicroRNA Targeting Specificity. Mol. Cell.

[21] Ha M., Kim V.N. (2014). Regulation of microRNA biogenesis. Nat. Rev. Mol. Cell Biol..

[22] O'Brien J., Hayder H., Zayed Y., Peng C. (2018). Overview of MicroRNA Biogenesis, Mechanisms of Actions, and Circulation. Front. Endocrinol..

[23] Paul S., Bravo Vázquez L.A., Pérez Uribe S., Roxana Reyes-Pérez P., Sharma A. (2020). Current Status of microRNA-Based Therapeutic Approaches in Neurodegenerative Disorders. Cells.

[24] Peplow P., Martinez B. (2021). MicroRNAs in laser-induced choroidal neovascularization in mice and rats: their expression and potential therapeutic targets. Neural Regen. Res..

[25] Jang S.Y., Yang J.Y., Park J.H., Kim Y., An S., Jung W.H., Park J.W., Han J.W., Kim J.H., Park H.S. (2025). The Role of the Mitogen-Activated Protein Kinase Pathway in the Development of Laser-Induced Choroidal Neovascularization. Int. J. Mol. Sci..

[26] Han J., Chang H., Yang J., Choi H., Park H., Jun H., Choi J., Paik S.S., Chung K., Shin H. (2023). Intravitreal Administration of Retinal Organoids-Derived Exosomes Alleviates Photoreceptor Degeneration in Royal College of Surgeons Rats by Targeting the Mitogen-Activated Protein Kinase Pathway. Int. J. Mol. Sci..

[27] Bernstein D.L., Jiang X., Rom S. (2021). let-7 microRNAs: Their Role in Cerebral and Cardiovascular Diseases, Inflammation, Cancer, and Their Regulation. Biomedicines.

[28] Yang J.Y., Choi J.S., Park T.K. (2026). AAV2.7m8-Mediated MicroRNA Expression Suppresses VEGF-Induced Angiogenic Responses in HUVEC. Int. J. Mol. Sci..

[29] Liao T.T., Hsu W.H., Ho C.H., Hwang W.L., Lan H.Y., Lo T., Chang C.C., Tai S.K., Yang M.H. (2016). let-7 Modulates Chromatin Configuration and Target Gene Repression through Regulation of the ARID3B Complex. Cell Rep..

[30] Oltra M., Vidal-Gil L., Maisto R., Oltra S.S., Romero F.J., Sancho-Pelluz J., Barcia J.M. (2019). miR302a and 122 are deregulated in small extracellular vesicles from ARPE-19 cells cultured with H(2)O(2). Sci. Rep..

[31] Gugliandolo A., Silvestro S., Sindona C., Bramanti P., Mazzon E. (2021). MiRNA: Involvement of the MAPK Pathway in Ischemic Stroke. A Promising Therapeutic Target. Medicina (Kaunas).

[32] Yang X., Liang L., Zhang X.F., Jia H.L., Qin Y., Zhu X.C., Gao X.M., Qiao P., Zheng Y., Sheng Y.Y. (2013). MicroRNA-26a suppresses tumor growth and metastasis of human hepatocellular carcinoma by targeting interleukin-6-Stat3 pathway. Hepatology.

[33] Faramin Lashkarian M., Hashemipour N., Niaraki N., Soghala S., Moradi A., Sarhangi S., Hatami M., Aghaei-Zarch F., Khosravifar M., Mohammadzadeh A. (2023). MicroRNA-122 in human cancers: from mechanistic to clinical perspectives. Cancer Cell Int..

[34] Xie P., Kamei M., Suzuki M., Matsumura N., Nishida K., Sakimoto S., Sakaguchi H., Nishida K. (2011). Suppression and regression of choroidal neovascularization in mice by a novel CCR2 antagonist, INCB3344. PLoS One.

[35] Dridi S., Hirano Y., Tarallo V., Kim Y., Fowler B.J., Ambati B.K., Bogdanovich S., Chiodo V.A., Hauswirth W.W., Kugel J.F. (2012). ERK1/2 activation is a therapeutic target in age-related macular degeneration. Proc. Natl. Acad. Sci. USA.

[36] Hua J., Guerin K.I., Chen J., Michán S., Stahl A., Krah N.M., Seaward M.R., Dennison R.J., Juan A.M., Hatton C.J. (2011). Resveratrol inhibits pathologic retinal neovascularization in Vldlr(-/-) mice. Investig. Ophthalmol. Vis. Sci..

[37] Chan C.M., Huang C.H., Li H.J., Hsiao C.Y., Su C.C., Lee P.L., Hung C.F. (2015). Protective effects of resveratrol against UVA-induced damage in ARPE19 cells. Int. J. Mol. Sci..

[38] Tsao Y.P., Ho T.C., Chen S.L., Cheng H.C. (2006). Pigment epithelium-derived factor inhibits oxidative stress-induced cell death by activation of extracellular signal-regulated kinases in cultured retinal pigment epithelial cells. Life Sci..

[39] Infante J.R., Fecher L.A., Falchook G.S., Nallapareddy S., Gordon M.S., Becerra C., DeMarini D.J., Cox D.S., Xu Y., Morris S.R. (2012). Safety, pharmacokinetic, pharmacodynamic, and efficacy data for the oral MEK inhibitor trametinib: a phase 1 dose-escalation trial. Lancet Oncol..

[40] Wilhelm S.M., Dumas J., Adnane L., Lynch M., Carter C.A., Schütz G., Thierauch K.H., Zopf D. (2011). Regorafenib (BAY 73-4506): a new oral multikinase inhibitor of angiogenic, stromal and oncogenic receptor tyrosine kinases with potent preclinical antitumor activity. Int. J. Cancer.

[41] Hu Y., Peng X., Du G., Zhang Z., Zhai Y., Xiong X., Luo X. (2022). MicroRNA-122-5p Inhibition Improves Inflammation and Oxidative Stress Damage in Dietary-Induced Non-alcoholic Fatty Liver Disease Through Targeting FOXO3. Front. Physiol..

[42] Wu M., Liu X., Li Z., Huang X., Guo H., Guo X., Yang X., Li B., Xuan K., Jin Y. (2021). SHED aggregate exosomes shuttled miR-26a promote angiogenesis in pulp regeneration via TGF-beta/SMAD2/3 signalling. Cell Prolif..

[43] Icli B., Wara A.K.M., Moslehi J., Sun X., Plovie E., Cahill M., Marchini J.F., Schissler A., Padera R.F., Shi J. (2013). MicroRNA-26a regulates pathological and physiological angiogenesis by targeting BMP/SMAD1 signaling. Circ. Res..

[44] Grimm D., Streetz K.L., Jopling C.L., Storm T.A., Pandey K., Davis C.R., Marion P., Salazar F., Kay M.A. (2006). Fatality in mice due to oversaturation of cellular microRNA/short hairpin RNA pathways. Nature.

[45] Khan A.A., Betel D., Miller M.L., Sander C., Leslie C.S., Marks D.S. (2009). Transfection of small RNAs globally perturbs gene regulation by endogenous microRNAs. Nat. Biotechnol..

[46] Grimm D., Wang L., Lee J.S., Schürmann N., Gu S., Börner K., Storm T.A., Kay M.A. (2010). Argonaute proteins are key determinants of RNAi efficacy, toxicity, and persistence in the adult mouse liver. J. Clin. Investig..

[47] Aagaard L.A., Zhang J., von Eije K.J., Li H., Sætrom P., Amarzguioui M., Rossi J.J. (2008). Engineering and optimization of the miR-106b cluster for ectopic expression of multiplexed anti-HIV RNAs. Gene Ther..

[48] Haldrup S.H., Fabian-Jessing B.K., Jakobsen T.S., Lindholm A.B., Adsersen R.L., Aagaard L., Bek T., Askou A.L., Corydon T.J. (2024). Subretinal AAV delivery of RNAi-therapeutics targeting VEGFA reduces choroidal neovascularization in a large animal model. Mol. Ther. Methods Clin. Dev..

[49] Yang J.Y., Madrakhimov S.B., Ahn D.H., Chang H.S., Jung S.J., Nah S.K., Park H.Y., Park T.K. (2019). mTORC1 and mTORC2 are differentially engaged in the development of laser-induced CNV. Cell Commun. Signal..

[50] Bolger A.M., Lohse M., Usadel B. (2014). Trimmomatic: a flexible trimmer for Illumina sequence data. Bioinformatics.

[51] Kim D., Paggi J.M., Park C., Bennett C., Salzberg S.L. (2019). Graph-based genome alignment and genotyping with HISAT2 and HISAT-genotype. Nat. Biotechnol..

[52] Pertea M., Pertea G.M., Antonescu C.M., Chang T.C., Mendell J.T., Salzberg S.L. (2015). StringTie enables improved reconstruction of a transcriptome from RNA-seq reads. Nat. Biotechnol..

[53] Robinson M.D., McCarthy D.J., Smyth G.K. (2010). edgeR: a Bioconductor package for differential expression analysis of digital gene expression data. Bioinformatics.

