# Supplementary material for: rAAV-mediated multi-miRNA delivery targeting MAPK pathway suppresses laser-induced choroidal neovascularization in mice
Source: Mol Ther Nucleic Acids. 2026 Sep 16;37(4):103101. doi: 10.1016/j.omtn.2026.103101 (PMC13651570; doi:10.1016/j.omtn.2026.103101)
Supplement: Supplementary file 1 [file mmc1.pdf]

OMTN, Volume 37

Supplemental information

rAAV-mediated multi-miRNA delivery targeting MAPK pathway suppresses laser-induced choroidal neovascularization in mice

Jung Woo Han, Jin Young Yang, Yeji Kim, Sumin An, Eunjae Yoo, In Hwan Park, Sang-Won Lee, Hyun-Ji Lee, Tae Geol Lee, Wook Hyun Jung, Hyo Song Park, Jin Ha Kim, Jun Sub Choi, Hun Soo Chang, and Tae Kwann Park

	MAPK related Genes										
	DUSP16	EGFR	FOS	MAP2K4	MAPK1	MAPK8	NF1	RASA2	RASGRP1	RPS6KA5	TEK
has-mir-122-5p		■					■				
has-let-7a-5p			■	■	■	■				■	■
has-mir-26a-5p			■			■		■	■		

Figure S1. Schematic representation of MAPK signaling pathway and target genes of selected miRNAs

The selected miRNAs—hsa-miR-122-5p, hsa-let-7a-5p, and hsa-miR-26a-5p—target multiple critical genes within these MAPK cascades, enabling simultaneous suppression of key pathological processes involved in AMD and CNV progression. Boxes indicate specific genes targeted by each miRNA, highlighting their comprehensive coverage of the MAPK pathway.

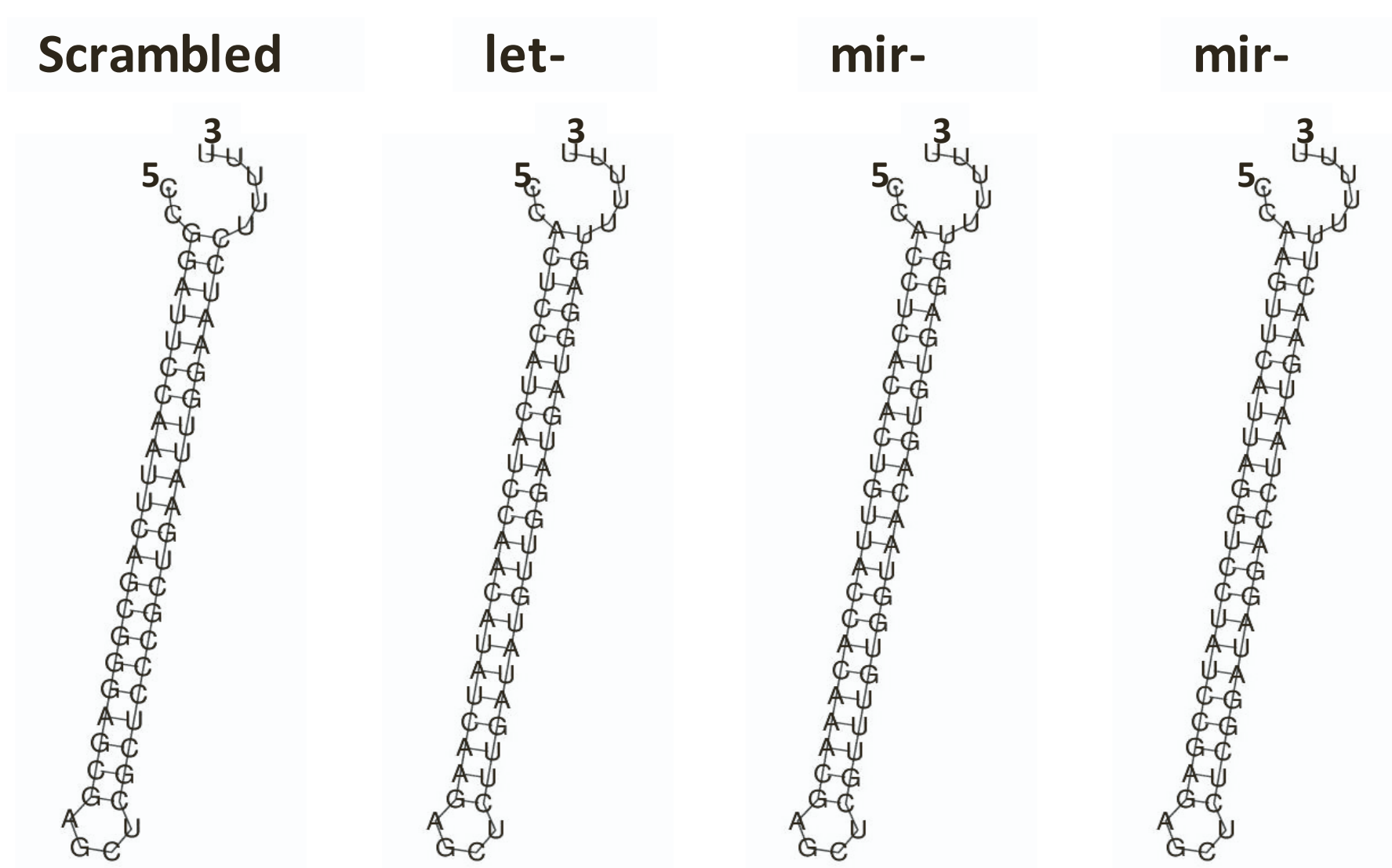

Figure S2. Predicted secondary structures of the shRNA constructs.

Predicted RNA secondary structures of the individual shRNA constructs used in this study are shown. The sequences are presented as RNA sequences, with uracil (U) instead of thymine (T), and are predicted to form characteristic stem-loop structures consistent with conventional shRNA architecture. The 5′ end of each RNA sequence is indicated.

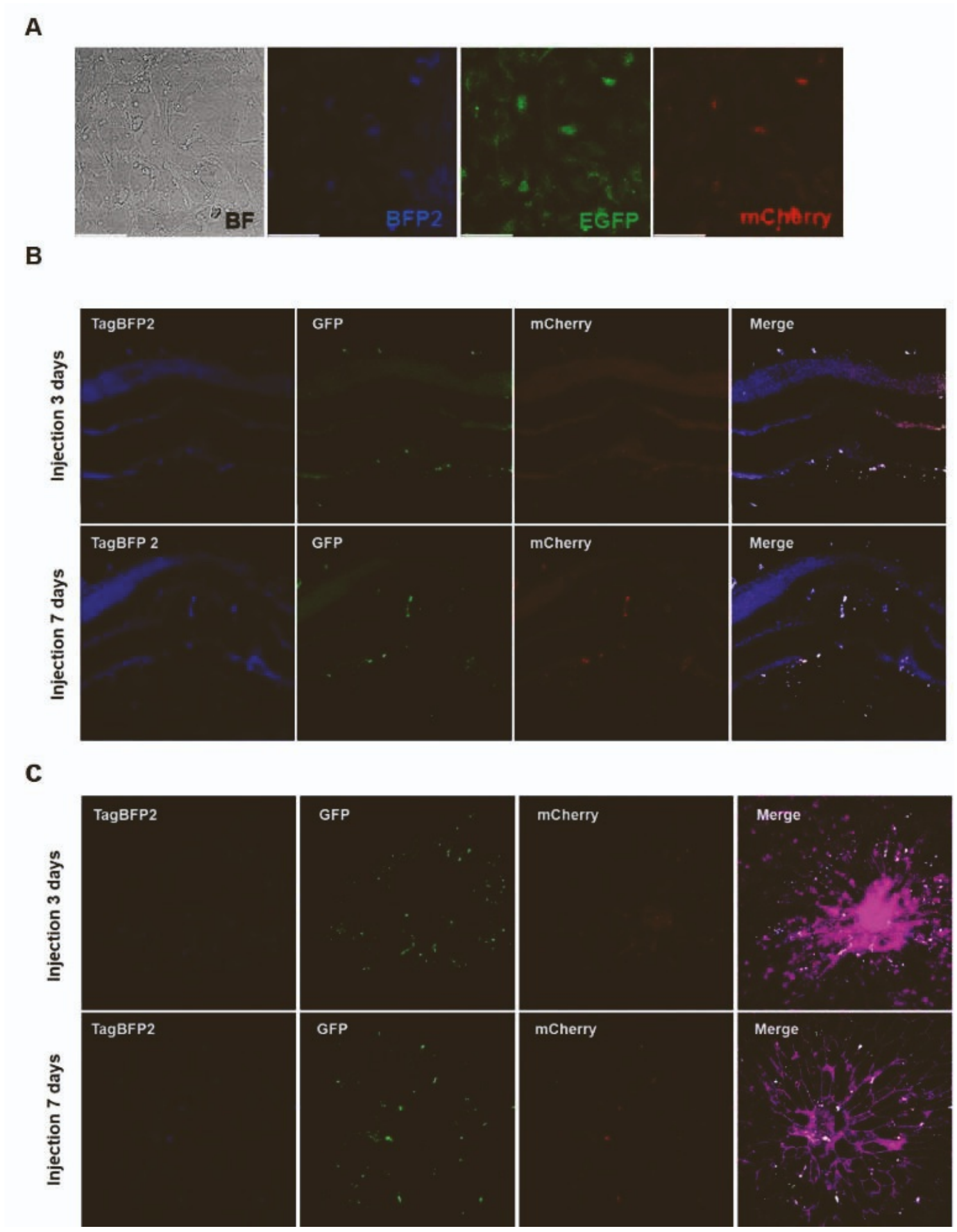

Figure S3. Transduction Efficiency of rAAV2.7m8-MAPK-miR constructs in vitro and in vivo.

(A) Representative fluorescence images of ARPE-19 cells following transduction with rAAV2.7m8 vectors expressing individual miRNA constructs. Bright-field (BF) and fluorescence channels (TagBFP2, EGFP, and mCherry) confirm successful expression of each construct in vitro. (B) Retinal cross-sections at 3 and 7 days after intravitreal injection showing expression of TagBFP2 (blue), EGFP (green), and mCherry (red). Merged images demonstrate co-localization and successful in vivo transduction of multiple constructs across retinal layers. (C) RPE–choroid flatmount images at 3 and 7 days post-injection. Fluorescence signals corresponding to TagBFP2, EGFP, and mCherry indicate effective delivery and expression within the RPE layer. Merged images demonstrate spatial distribution and co-localization of the three constructs. Scale bar: 100 μm

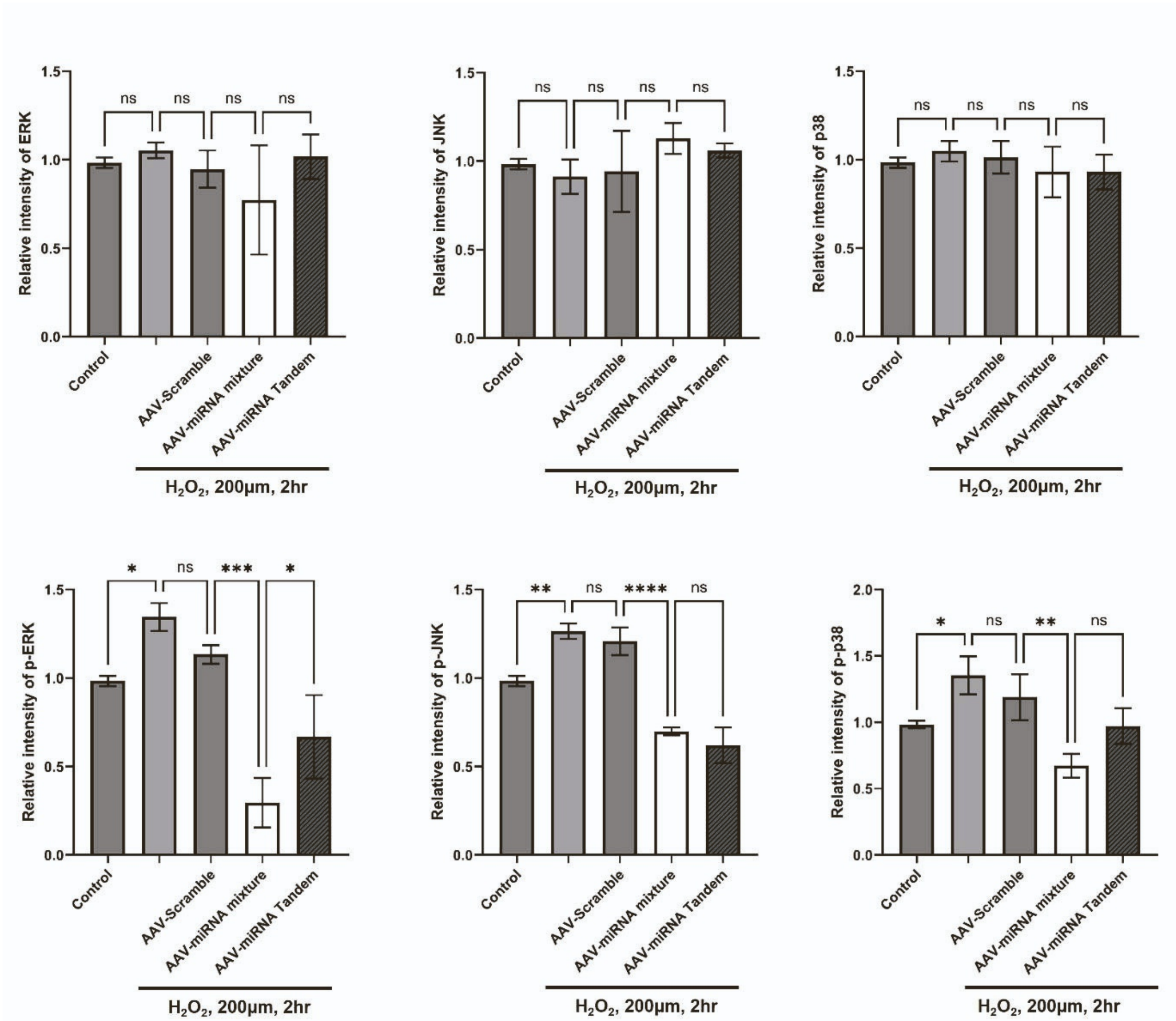

Figure S4. Quantitative analysis of total and phosphorylated MAPK pathway proteins in H_2O_2-treated ARPE-19 cells.

ARPE-19 cells were exposed to H_2O_2 to induce oxidative stress, followed by treatment with rAAV2.7m8-MAPK-miR mixture or rAAV2.7m8-MAPK-miR tandem. (A) analyses of total ERK, JNK, and p38 protein levels demonstrated no significant differences among the experimental groups. (B) In contrast, the levels of phosphorylated MAPK proteins, including p-ERK, p-JNK, and p-p38, were markedly reduced in the rAAV2.7m8-MAPK-miR mixture- and tandem-treated groups compared with the H_2O_2-treated control group. Consistent with these findings, the phosphorylation ratios (p-ERK/ERK, p-JNK/JNK, and p-p38/p38) also showed a similar pattern of reduction in the mixture- and tandem-treated groups. Data are presented as mean ± SEM. Statistical analysis was performed using one-way ANOVA followed by Tukey's multiple comparisons test.

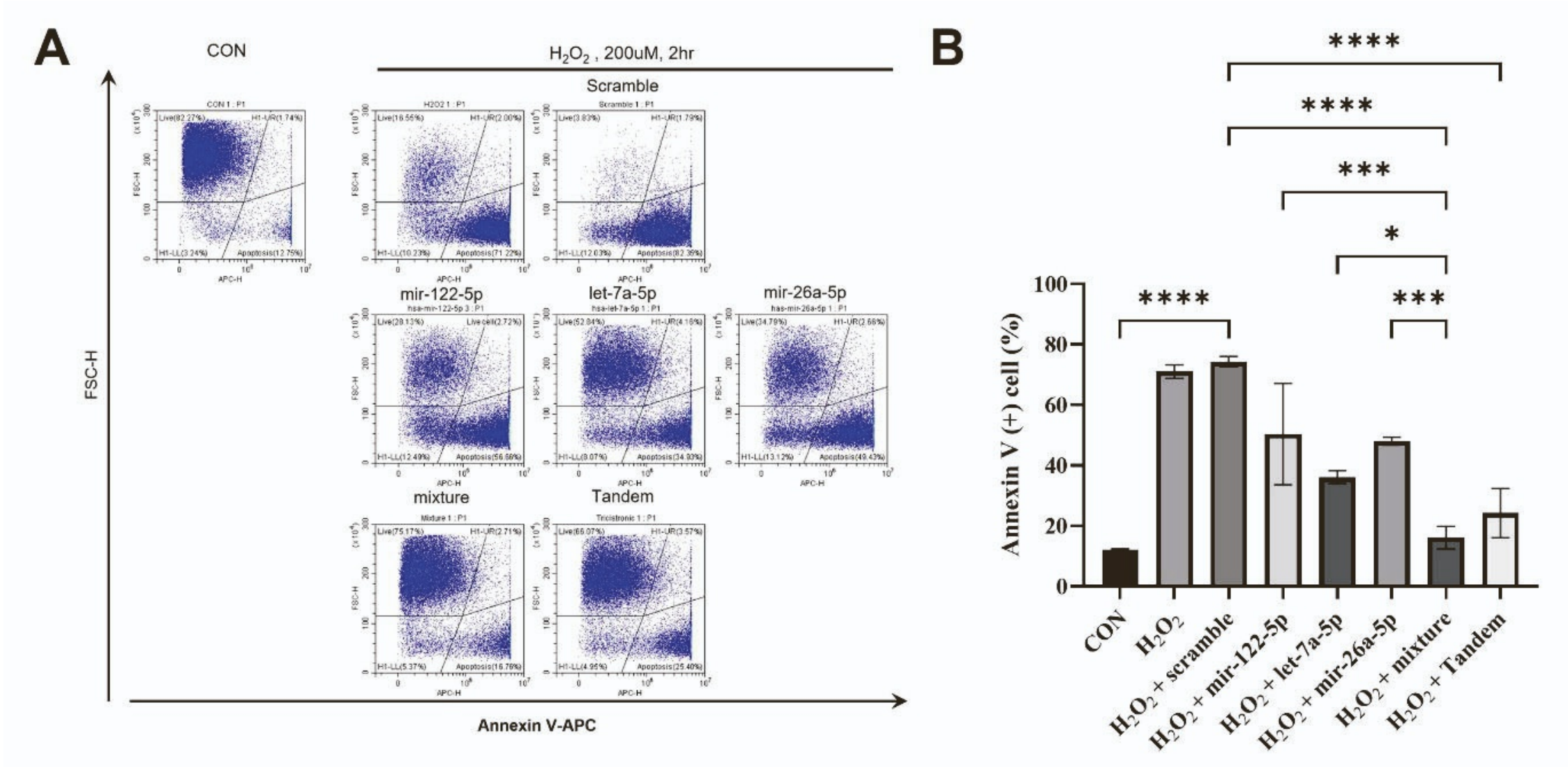

Figure S5. Comparative analysis of apoptosis in ARPE-19 cells treated with individual and multi-miRNA constructs under oxidative stress.

(A) Representative flow cytometry plots showing Annexin V-APC staining for apoptosis detection in ARPE-19 cells treated with 200 μM H_2O_2 for 2 hours, followed by individual miRNAs (hsa-miR 122-5p, hsa-let-7a-5p, and hsa-miR-26a-5p), rAAV-miRNA mixture (AAV-mixture), or rAAV-miRNA tandem (AAV-Tandem). (B) Quantitative analysis of Annexin V-positive apoptotic cells (%). The rAAV miRNA mixture showed significantly greater reduction in apoptosis compared to individual miRNA and tandem constructs. Data are presented as mean ± SEM from at least three independent experiments. Statistical analysis was performed using one-way ANOVA followed by Tukey's multiple comparisons test. *$p < 0.05$, ***$p < 0.001$, ****$p < 0.0001$; ns, not significant.

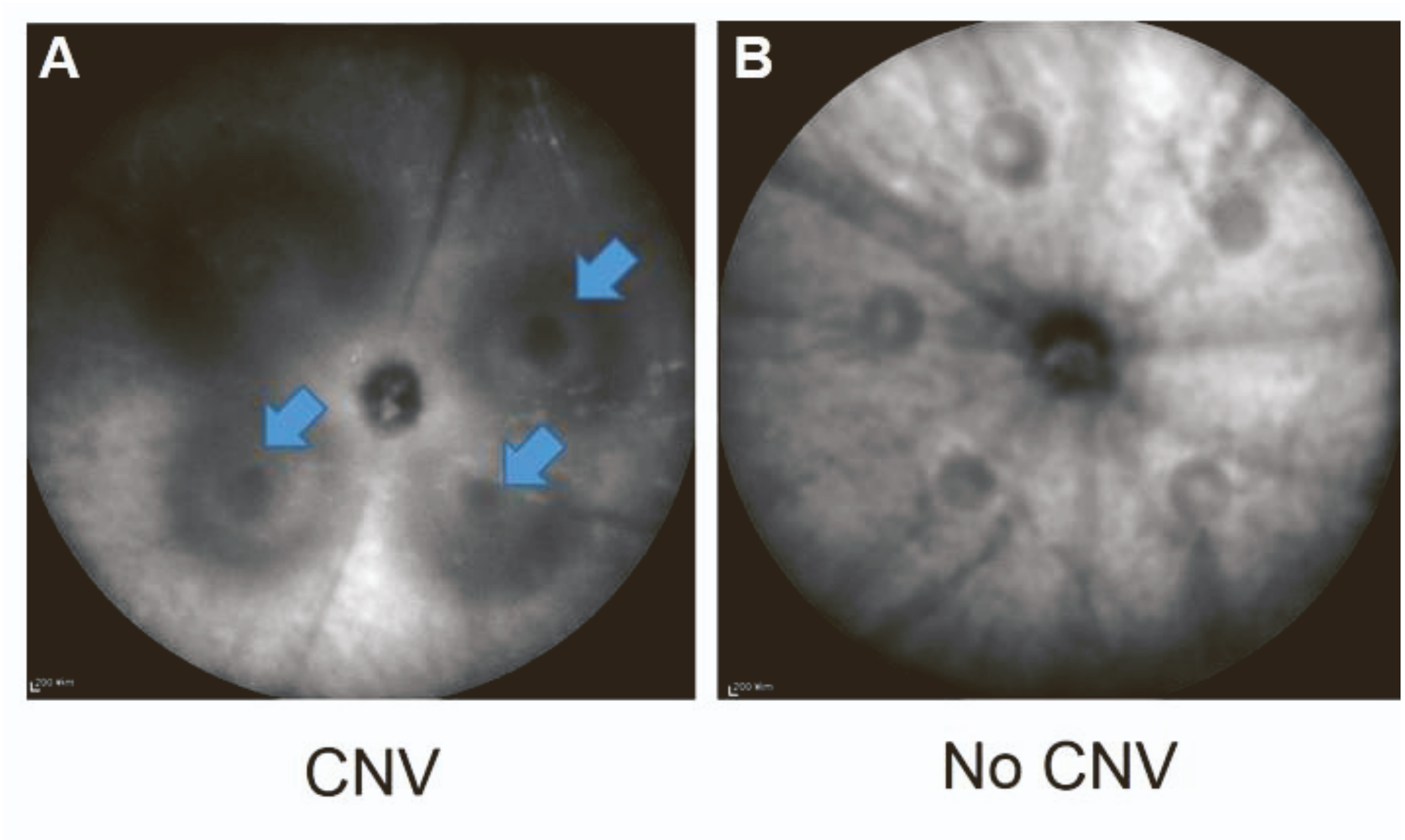

Figure S6. Validation of laser-induced CNV lesions by infrared (IR) imaging.

(A) Laser burns showing characteristic bubble formation (arrows), indicating rupture of Bruch's membrane and successful induction of choroidal neovascularization (CNV). (B) Laser burns without bubble formation, representing intact Bruch's membrane and failure to induce CNV. These lesions were excluded from subsequent analysis.

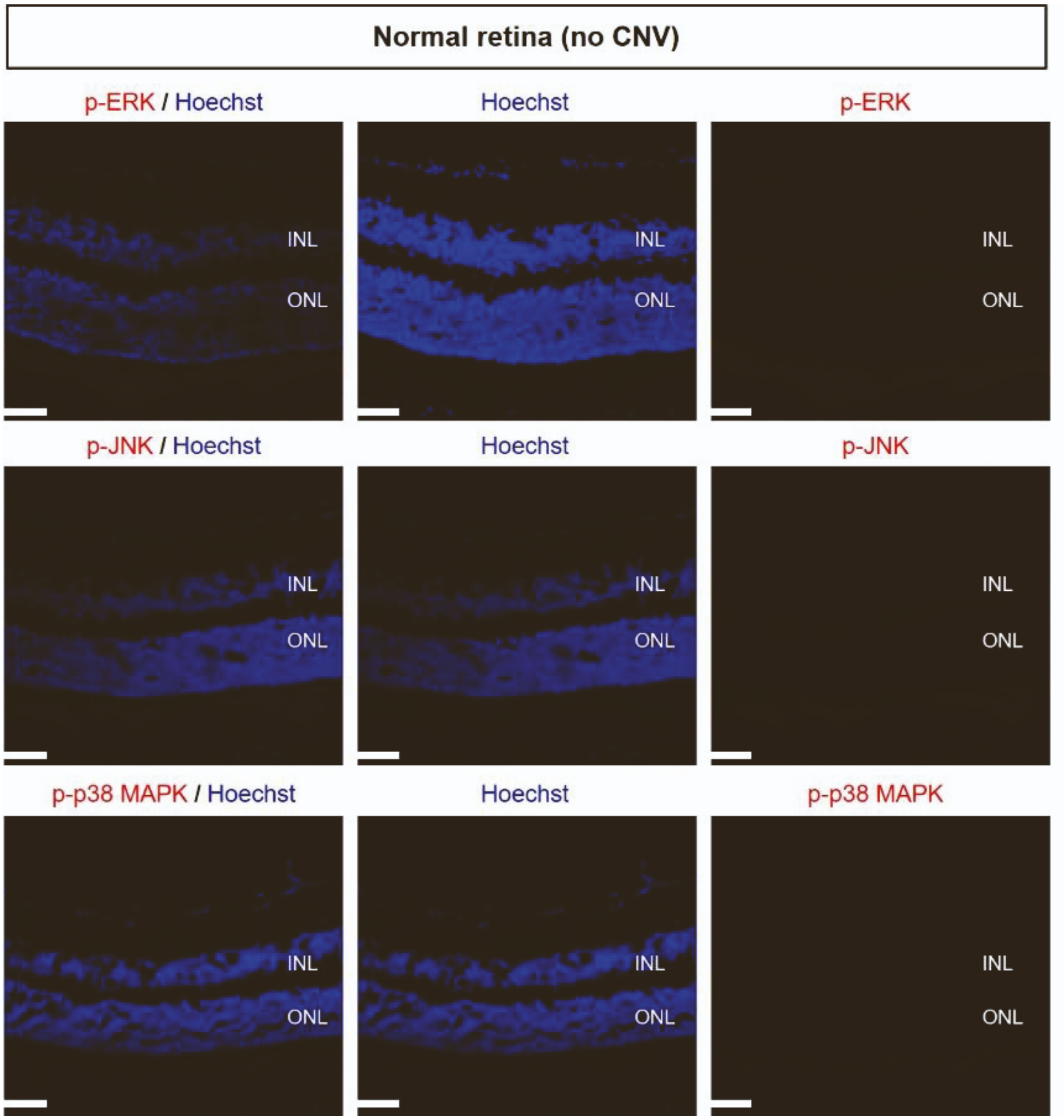

Figure S7. Minimal activation of MAPK signaling in the normal retina.

Representative immunofluorescence images of phosphorylated MAPK proteins in normal (non-CNV) mouse retina. Phosphorylated ERK (p-ERK), JNK (p-JNK), and p38 MAPK (p-p38) are shown in red, with nuclear counterstaining by Hoechst (blue). In the absence of laser-induced injury, all three phosphorylated MAPK signals were minimally detected across retinal layers, including the inner nuclear layer (INL) and outer nuclear layer (ONL), indicating low basal MAPK activity under physiological conditions. Scale bar: 100 μm

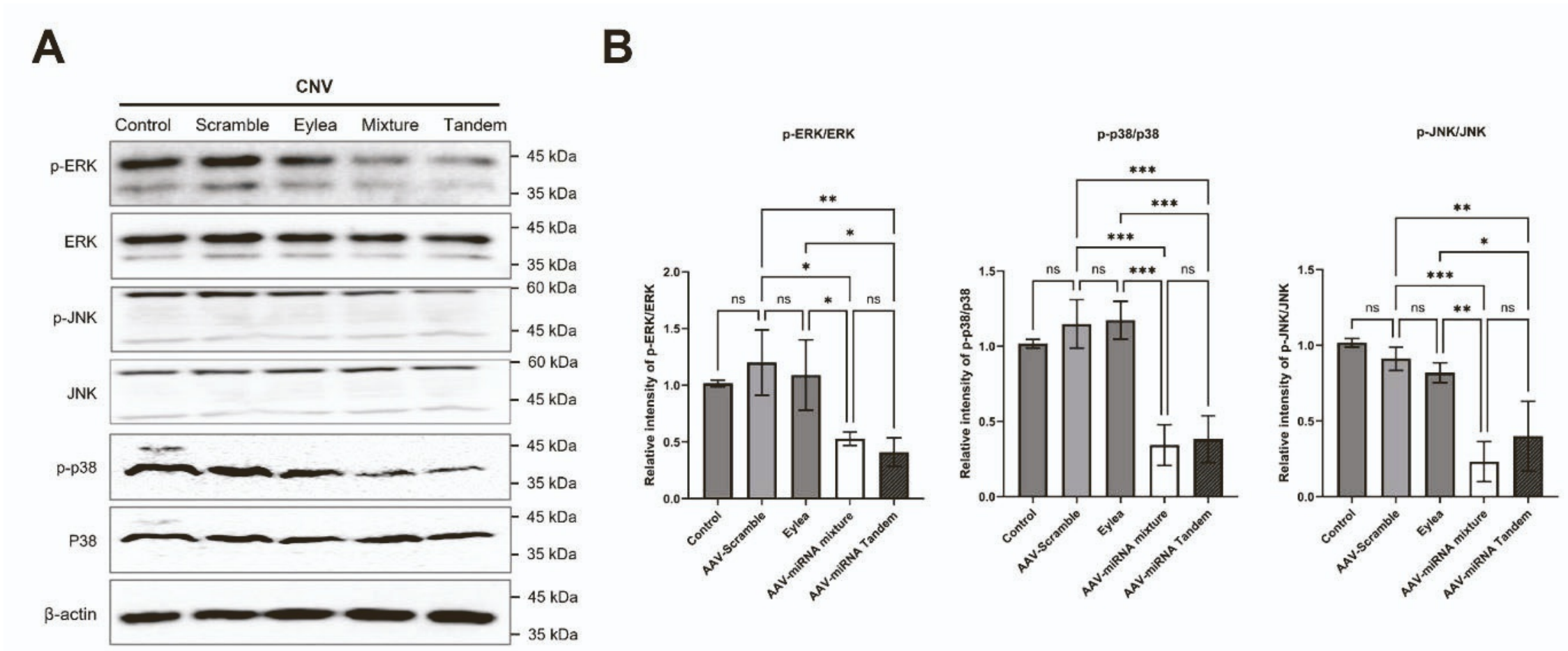

Figure S8. rAAV2.7m8-MAPK-miR treatment suppresses MAPK signaling in vivo.

(A) Representative Western blot images showing the expression of phosphorylated and total MAPK proteins, including ERK (p-ERK/ERK), JNK (p-JNK/JNK), and p38 (p-p38/p38), in retinal tissues from different treatment groups: control, AAV-scramble, aflibercept, rAAV2.7m8-MAPK-miR mixture, and rAAV2.7m8-MAPK-miR tandem. β-actin was used as a loading control. (B) Quantitative analysis of phosphorylated MAPK proteins normalized to their respective total protein levels (p-ERK/ERK, p-JNK/JNK, and p-p38/p38). Both rAAV2.7m8-MAPK-miR mixture and tandem treatments significantly reduced MAPK activation compared to control and AAV-scramble groups. Aflibercept showed a limited effect on MAPK signaling. Data are presented as mean ± SEM. Statistical analysis was performed using one-way ANOVA followed by Tukey's multiple comparisons test. (*$p < 0.05$, ***$p < 0.001$, ****$p < 0.0001$; ns, not significant)

Table S1. Raw Ct values for miRNA expression analysis presented in Figure 1C.

Raw cycle threshold (Ct) values obtained by quantitative real-time PCR for hsa-miR-122-5p, hsa-let-7a-5p, and hsa-miR-26a-5p are presented for all experimental groups, including the No AAV, AAV-miRNA mixture, and AAV Tandem groups. Raw Ct values from three independent samples (n = 3) are provided for each experimental group.

CT raw data (n=3)													
hsa-mir-122-5p													
1'	No AAV	AAV-miRNA mixtures	AAV Tandem		2'	No AAV	AAV-miRNA mixtures	AAV Tandem		3'	No AAV	AAV-miRNA mixture	AAV Tandem
n=1	39.44	28.82	30.15		n=1	39.99	29.46	29.85		n=1	40	29.8	29.9
n=2	39.55	29.585	30.633		n=2	39.99	29.95	30.19		n=2	40	29.954	30.518
n=3	39.66	29.827	30.766		n=3	39.995	30.679	30.719		n=3	40	30.46	31.054
hsa-let-7a-5p													
1'	No AAV	AAV-miRNA mixtures	AAV Tandem		2'	No AAV	AAV-miRNA mixtures	AAV Tandem		3'	No AAV	AAV-miRNA mixture	AAV Tandem
n=1	39.98	33.98	34.88		n=1	39.82	34.55	35.47		n=1	39.84	33.72	34.85
n=2	39.99	34.38	35.37		n=2	39.936	34.87	35.77		n=2	39.9	33.948	34.854
n=3	39.99	34.64	35.72		n=3	39.88	35.33	36.05		n=3	39.94	34.572	35.62
hsa-let-7a-5p													
1'	No AAV	AAV-miRNA mixtures	AAV Tandem		2'	No AAV	AAV-miRNA mixtures	AAV Tandem		3'	No AAV	AAV-miRNA mixture	AAV Tandem
n=1	39.91	31.75	32.88		n=1	39.9	30.85	33.05		n=1	39.955	30.35	31.75
n=2	39.98	31.845	33.55		n=2	39.95	31.066	33.27		n=2	39.975	30.846	31.856
n=3	39.995	32.389	33.768		n=3	39.92	31.683	33.54		n=3	39.97	31.743	32.474
